# nNOS-derived NO modulates force production and iNO-derived NO the excitability in C2C12-derived 3D tissue engineering skeletal muscle *via* different NO signaling pathways

**DOI:** 10.3389/fphys.2022.946682

**Published:** 2022-08-15

**Authors:** Matias Mosqueira, Lisa-Mareike Scheid, Dominik Kiemel, Talisa Richardt, Mona Rheinberger, Dirk Ollech, Almut Lutge, Tim Heißenberg, Lena Pfitzer, Lisa Engelskircher, Umut Yildiz, Isabel Porth

**Affiliations:** ^1^ Cardio-Ventilatory Muscle Physiology Laboratory, Institute of Physiology and Pathophysiology, Heidelberg University Hospital, Heidelberg, Germany; ^2^ PromoCell GmbH, Heidelberg, Germany; ^3^ Department of Infectious Diseases, Centre for Integrative Infectious Disease Research (CIID), Heidelberg University, Heidelberg, Germany; ^4^ Applied Physics Department, Science for Life Laboratory and KTH Royal Technical University, Solna, Sweden; ^5^ Department of Molecular Life Science at the University of Zürich, Zürich, Switzerland; ^6^ Institute of Organic and Biomolecular Chemistry, Georg-August-Universität, Göttingen, Germany; ^7^ myNEO NV, Ghent, Belgium; ^8^ Immatics Biotechnology GmbH, Tübingen, Germany; ^9^ European Molecular Biology Laboratory, Genome Biology Unit, Heidelberg, Germany; ^10^ Institute of Pathology, University Medical Centre Mannheim, University of Heidelberg, Mannheim, Germany

**Keywords:** C2C12, tissue engineering, nitric oxide, calcium, force, myooid

## Abstract

Nitric oxide (NO) is a bioactive gas produced by one of the three NO synthases: neuronal NOS (nNOS), inducible (iNOS), and endothelial NOS (eNOS). NO has a relevant modulatory role in muscle contraction; this takes place through two major signaling pathways: (i) activation of soluble guanylate cyclase and, thus, protein kinase G or (ii) nitrosylation of sulfur groups of cysteine. Although it has been suggested that nNOS-derived NO is the responsible isoform in muscle contraction, the roles of eNOS and iNOS and their signaling pathways have not yet been clarified. To elucidate the action of each pathway, we optimized the generation of myooids, an engineered skeletal muscle tissue based on the C2C12 cell line. In comparison with diaphragm strips from wild-type mice, 180 myooids were analyzed, which expressed all relevant excitation–contraction coupling proteins and both nNOS and iNOS isoforms. Along with the biochemical results, myooids treated with NO donor (SNAP) and unspecific NOS blocker (L-NAME) revealed a comparable NO modulatory effect on force production as was observed in the diaphragm strips. Under the effects of pharmacological tools, we analyzed the myooids in response to electrical stimulation of two possible signaling pathways and NO sources. The nNOS-derived NO exerted its negative effect on force production *via* the sGG-PKG pathway, while iNOS-derived NO increased the excitability in response to sub-threshold electrical stimulation. These results strengthen the hypotheses of previous reports on the mechanism of action of NO during force production, showed a novel function of iNOS-derived NO, and establish the myooid as a novel and robust alternative model for pathophysiological skeletal muscle research.

## Introduction

Muscle force production is an excitation–contraction coupling-dependent process triggered by the motor neuron action potential, activating the dihydropyridine-sensitive calcium voltage-gated channel (DHPR), which in turn opens the ryanodine receptor (RyR1) and releases calcium (Ca^2+^) from the sarcoplasmic reticulum (SR). Consequently, Ca^2+^ binds to troponin-C, resulting in the movement of tropomyosin and the interaction of myosin heads with actin filaments, resulting in shortening of the sarcomere. These events lead to the contraction of the muscle, measured as force production. This process is mainly regulated by motor neuron activity and Ca^2+^ release, but other signaling pathways also modulate force production. For instance, different models have shown that, under physiological conditions, NO negatively modulates force production (Reid, 1998; Li et al., 2011; Xiyuan et al., 2017), although there are reports demonstrating that these effects are dependent on the partial pressure of oxygen in solution (Eu et al., 2000; Eu et al., 2003). Nanomolar concentrations of bioactive gas NO produced with a short half-life molecule (Ignarro et al., 1987) bind tightly to a prosthetic heme on the β-subunit of soluble guanylyl cyclase (sGC), increasing the conversion of GTP to cGMP from 100- to 200-fold (Francis et al., 2010). This results in activation of PKG, cGMP-cation gated channels, cGMP-hydrolyzing phosphodiesterases (PDE), and cGMP-binding cGMP-hydrolyzing PDEs, leading to modification of several physiological processes (Francis et al., 2010). In skeletal muscle, the cGMP-PKG pathway negatively modulates contractility, accelerates relaxation, and improves the stiffness of myocytes *via* direct PKG phosphorylation targets, such as troponin-I, DHPR, phospholamban, Titin, inositol triphosphate receptor, Na^+^/K^+^-ATPase α-subunit, PDE5, ryanodine receptor, and transient receptor potential Ca^2+^ channel isoform 3 and 6 (Francis et al., 2010; Xiyuan et al., 2017). Alternative NO pathways, such as S-nitrosylation (SNO), result from the interaction of oxidized NO into peroxynitrite with free reactive cysteine thiol groups to form S-nitrosothiols (Anzai et al., 2000; Eu et al., 2000; Stamler and Meissner, 2001; Sun et al., 2001) and the nitration of tyrosine rings (Viner et al., 1999; Squier and Bigelow, 2000; Uda et al., 2020). NO is synthesized endogenously from L-arginine by catalytic action and upon specific stimuli of NO synthase (NOS) (Knowles and Moncada, 1994; Nathan and Xie, 1994). NOS isoforms have been described in mammalian cells, named after the cells or systems where they were first purified. Neuronal NOS (nNOS or NOS1) and endothelial NOS (eNOS or NOS3) are constitutively expressed and activated by Ca^2+^/calmodulin, while inducible NOS (iNOS or NOS2) is Ca^2+^ independent and enhances its activity and expression in response to bacterial endotoxin and inflammatory cytokines (Knowles and Moncada, 1994; Nathan and Xie, 1994). In skeletal muscle fibers, nNOS is located in cytoskeletal structures or plasma membranes through binding to the dystrophin-associated protein, α1-syntrophin (Wakayama et al., 1997) and is highly concentrated at the inner surface of the sarcolemma, subsarcolemmal areas near neuromuscular junctions, and myotendinous junctions and costameres (Chang et al., 1996; Grozdanovic and Gossrau, 1998). The expression of iNOS is found on the cystosolic side of the sarcoplasm in post-natal and adult mice and rats (Blottner and Luck, 1998; Patel et al., 2004), in the differentiated myotube L6E9 rat cell line (Kaliman et al., 1999), and in the 5-day differentiated myotube C2C12 mouse cell line (Blottner and Luck, 1998; Williams et al., 2006). The first reports showed that iNOS expression was enhanced only under certain inducers, including insulin growth factor-II (Kaliman et al., 1999), transforming growth factor β-1 (Williams et al., 2006), NF-κB (Lee et al., 1997), or ischemia-reperfusion protocols (Patel et al., 2004), but lately it has been shown that iNOS is also expressed without any external stimuli (Ham et al., 2015). eNOS is localized close to caveoline-3 in the sarcoplasm, close to mitochondria, capillaries, and arterioles, suggesting a role in the regulation of oxidative phosphorylation and blood flow in the skeletal muscle (Segal et al., 1999; Buchwalow et al., 2005). Although NO plays a relevant modulatory role in muscle contraction, it remains unknown how its action, through signaling pathways, modulates production and the contribution of each isoform to force production.

Elucidating the precise effect of the NO-GC-PKG or NO-S-nitrosylation pathways and the NO source during muscle contraction would require a large number of animals. To overcome this ethical issue, we developed a muscle fiber based on tissue engineering (TE) techniques using a murine skeletal muscle cell line, C2C12. TE skeletal muscle not only diversifies the research field by producing muscle fibers from different sources of skeletal muscle, satellite cells, or cell lines (Fernandez-Costa et al., 2021; Khodabukus, 2021), but also reduces the number of animals and the high cost and time associated with producing mutations or transgenes for diverse muscle pathologies. Leading innovation in this field, Dennis and Kosnik (2000) (Dennis et al., 2001; Kosnik et al., 2001) developed a three-dimensional skeletal muscle construct organoid termed a myooid by co-culturing rat soleus satellite cells and fibroblasts in the center region without the use of an artificial scaffold. Later, Dennis et al. further developed the technique by comparing different sources of skeletal muscle and the C2C12 skeletal muscle cell line using classical muscle parameters (Dennis et al., 2001). TE in skeletal muscle has been broadly applied to successfully evaluate technical (cell source, media, serum, electrical stimulation, scaffold, coating, and growth factors) morphological, biochemical, histological, and physiological aspects of the 3D muscle model (Park et al., 2008; Fujita et al., 2009; Boonen et al., 2010; Fujita et al., 2010; Nagamine et al., 2010; Langelaan et al., 2011; Sato et al., 2011; Shimizu et al., 2013; van der Schaft et al., 2013; Khodabukus et al., 2015; Khodabukus, 2021). Whereas these studies helped in the progress of TE in skeletal muscle, the consequences of these factors on the physiology of muscle contraction or its regulation were mostly neglected.

In the current study, we optimized strategies to produce C2C12 based myooids for elucidating excitability and force production parameters *via* electrical stimulation-trigger. The physiological role of NO on parameters of muscle contraction obtained from diaphragm muscle strips was compared to those observed in myooids. The efficient strategy to produce myooids was then validated by utilizing it to precisely the mechanism of action by which the NO signaling pathway would modulate the force production in skeletal muscle. Although myooids produce a smaller force than adult murine diaphragm muscle strips, we observed that they had a similar shape and response to electrical stimulation as well as the effect of NO donor SNAP or NOS blocker L-NAME. Moreover, this study showed that the modulatory effect of NO on EC-coupling in myooids was analogous to previous reports and observations here in the diaphragm, and further demonstrated that nNOS-derived NO acted *via* the GC-PKG signaling pathway. Interestingly, the myooids revealed a new function of the iNOS-derived NO as a modulator of excitability to sub-threshold electrical stimulation through peroxynitrite production, which results in S-nitrosylation of thiol groups or nitration of tyrosine rings.

## Materials and methods

### Cell culture

All medium was based on Dulbecco’s Modified Eagle’s Medium-high glucose (DMEM-HG cat no. D5796, Sigma-Aldrich). Components added to the medium were sterile filtered with syringe filters with a 0.45 µm pore size (GE Healthcare, Whatman). The myoblast cell line C2C12 (DSMZ, ACC 565) was maintained at 37°C in 5% CO_2_ at 95% humidity in growth medium (GM) containing DMEM-HG (Sigma-Aldrich), 10% fetal bovine serum (F6178, Sigma-Aldrich), and 1% penicillin/streptomycin (15140122, Thermo Fisher Scientific). Differentiation medium (DM) was prepared from DMEM-HG with 2% horse serum (H1138, Sigma-Aldrich) and 1% penicillin/streptomycin.

### Myooid formation

**FIGURE 1:**
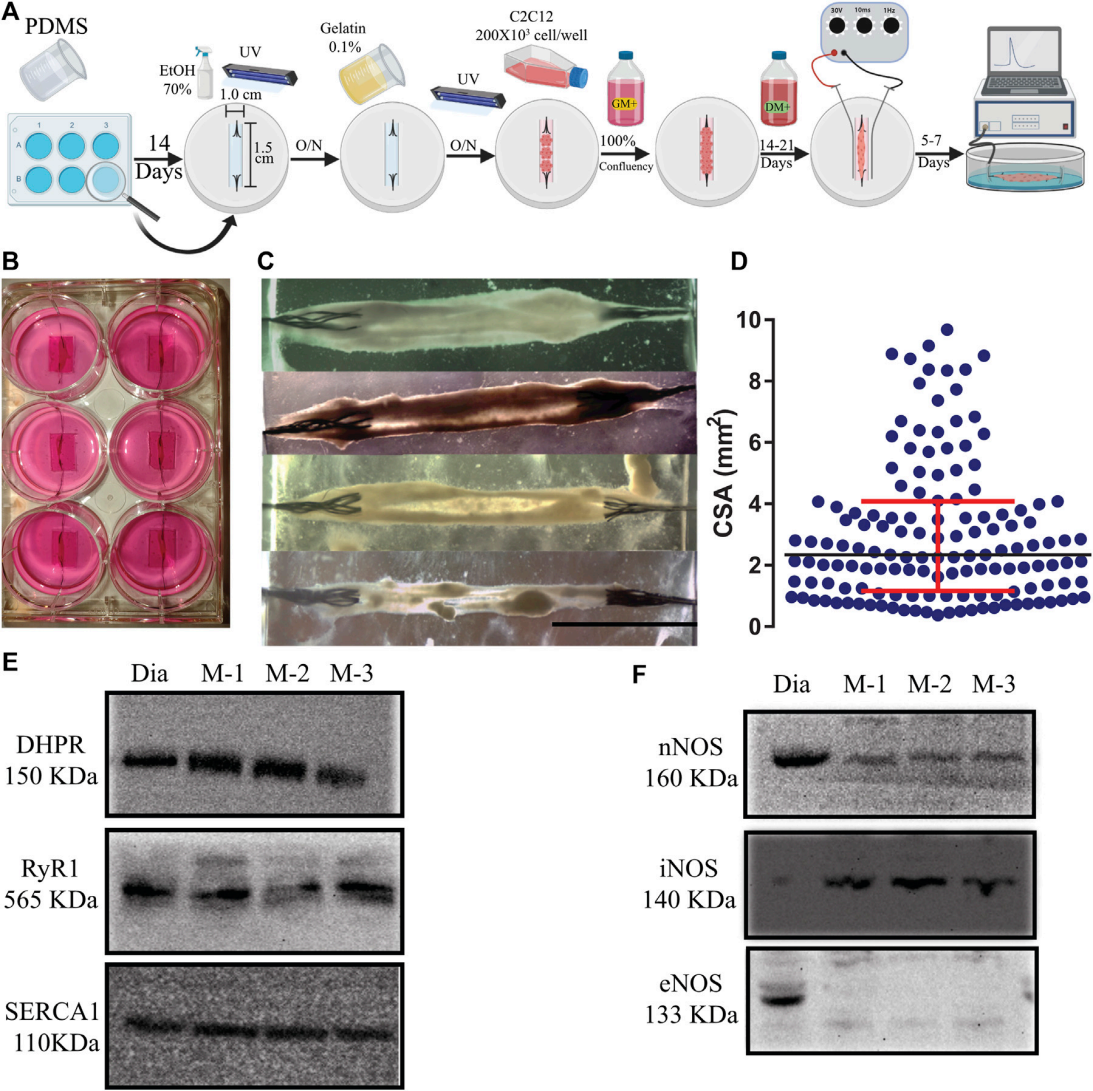
**(A)**. Schematic representation of the 3D tissue-engineering of myooids from the C2C12 cell line. A time course is described from left to right (O/N = overnight). Details of the protocol are described in Material and Methods. **(B)** Photography of a 6-well plate with six well-formed myooids after 21 days under differentiation medium. **(C)** Photomicrography of four different myooids just before setting them up on the force transducer. The bar represents 0.5 cm. **(D)** Data analysis with the representation of the cross-sectional area of 180 myooids analyzed in this study. E and **(F)** Images of six immunoblottings from diaphragm muscle (Dia) and three different pools of five myooids each (M-1, M-2, and M-3). The molecular weight is described under the abbreviation of the name of each protein. **(E)** Immunoblotting images for DHPR, RyR1, and SERCA1. **(F)** Immunoblotting images for nNOS, iNOS, and eNOS.

Schematization of the protocol for myooid formation is shown in Figure 1A. Two ml of polydimethylsiloxane layers (PDMS, curing agent: prepolymer 1:10; Sylgard 184, Dow Corning) were added to each of the wells of a 6-well plate and cured for a minimum of 14 days. After curing time, a well of 1.5 cm × 1.0 cm was carved until the bottom with two incisions in the long axis. A 1.0 cm-long suture (silk sutures, 0.7 metric H1F; RESORBA) was fringed at one end and fixed in each incision as artificial tendons. The 6-well plate was then sprayed with ethanol 70%, treated with UV light for 20 min, and air-dried in laminar flow overnight. Each well was coated with gelatin (0.1%; G1393, Sigma-Aldrich), air-dried in laminar flow overnight and UV light for 20 min. Once finalized, the plates were incubated at 37°C, 5% CO_2_ with GM containing gelatin (0.1%) for 5–7 days to control possible contamination. For myooid-formation, 200 × 10^3^ C2C12 myoblasts were seeded into each well and kept in GM+ (GM supplemented with 1 mM sodium pyruvate; S8636, Sigma-Aldrich) until confluence. Consecutively, myoblast differentiation was induced with DM+ (DM containing 1 mM sodium pyruvate and 10 ng/ml IGF, I1146, Sigma-Aldrich). Media were changed with 2–3 ml every other day.

Myooids were considered formed once the 2-D cell layer detached, rolled up and anchored to both sutures, which usually occurred spontaneously (Figures 1B,C). When cell layers had not detached from the surface at day 20 after seeding, the process was accelerated with the help of micropipette-tips. Electrical pulse stimulation (EPS) was given using a custom-built lid with two platinum electrodes (0.25 mm in diameter; Sigma-Aldrich) per well flanking each myooid. Formed myooids were stimulated for up to 10 days in DM+ with bipolar pulses at 1 Hz with 10 ms duration and 30 V amplitude, while the medium was changed every day.

### Cross-sectional area

Since the thickness dimension of the produced myooids was smaller than the short axis, it was not possible to assume that the myooid was cylindrical in shape. To resolve this issue, a new strategy was designed to calculate the CSA. Three different measurements in the short axis from the 2D images were taken and used to average the CSA in each myooid (Figure 1D).

### Force measurement for the myooid and diaphragm

All experiments were approved by the ethics committee of the University of Heidelberg Interfaculty Biomedical Research Facility (T-83/14) and conducted according to the guidelines of the Regierungspräsidium Karlsruhe of the State of Baden- Wuerttemberg. Diaphragm strips were harvested from the medial region of 6-month-old male C57Bl10 mice. As described elsewhere, the central tendon and the ribs attached to the diaphragm were set in the force transducer as the formula for normalizing diaphragm force production to CSA (Moorwood et al., 2013). CSA was used as described previously (Moorwood et al., 2013). All experiments were performed in a carbogen-bubbled Ringer-Krebs-Hensenleit (RKH) solution (composition in mM): NaCl 140, KCl 5, MgCl_2_*6H_2_O 1.2, CaCl_2_*2H_2_O 2, KH_2_PO_4_ 1.2, HEPES 20, pH 7.40 with NaOH, where glucose 6 and L-Arginine 0.2 were freshly added. Force measurements were performed with a Myoscope setup (Myotronic Heidelberg) carrying the force transducer TR 5 S (1.5 µN - 0.25 N) for myooids and TR7S for diaphragm strip (0–800 mN). The setup with a fixed myooid or diaphragm strip was mounted onto an Olympus Second Generation OSP-3 system. Myooids and diaphragm strips were placed in a cuvette with carbogen-bubbled RKH solution at 20°C flanked by two platinum electrodes for electrical field stimulations that were controlled with the Myodat software and a stimulator (both Myotronic Heidelberg). Between measurements, the myooid or diaphragm was perfused with a carbogen-bubbled RKH solution. Force responses were A/D converted with the PowerLab 4/35 (ADInstruments) and recorded with the software LabChart- Pro V8 (AD Instruments). The optimal length (Lo) was determined with a single twitch stimulus (50 V, 20 ms) under adjustment of the myooid length until the maximal force was reached. The excitability parameters known as rheobase and chronaxie are defined as the minimum amplitude of a stimulus at 100 ms pulse duration leading to a response of the muscle and the minimal pulse duration at twice the rheobase leading to a response, respectively. To determine these parameters, the myooid was stimulated with single pulses at predefined combinations of pulse duration and amplitude. The force-frequency relation was recorded at 50 V and 5 ms pulse duration during 500 ms with increasing frequencies (1, 5, 10, 15, 20, 25, 30, 35, 40, 45, 50, 60 Hz) in 2 min intervals with constant perfusion.

### Pharmacological agents

All pharmacological agents were purchased from Sigma-Aldrich if not stated otherwise and freshly dissolved in RKH to a working concentration. After establishing the optimal length (Lo) in control RKH, the myooid was perfused with a pharmacological agent for 15–20 min until the measurements were performed as described before. Working concentration used were 100 µM NO-donor SNAP (S-Nitroso-N-acetyl-DL-penicillamine; N3398); 5 mM NO-synthase inhibitor L-NAME (N_ω_-Nitro-L-arginine methyl ester; N5751); 100 µM soluble guanylyl cyclase inhibitor ODQ (1H-[1,2,4]Oxadiazolo[4,3-a]quinoxalin-1-one; O3636); 100 µM soluble guanylyl cyclase activator Bay41-2272 (B8810); 10 µM selective PKGI activator 8-pCPT-cGMP (8-(4-Chlorophenylthio)-guanosine 3′,5′-cyclic monophosphate sodium salt; C5438); 100 nM selective PKGI inhibitor DT-3 (Calbiochem, 370655). S-nitrosylation and nitration *via* peroxynitrite were blocked with 100 µM NEM (N-ethylmaleimide; E3876) or 1 mM ascorbic acid (A5960). Specific nNOS blocker 100 nM SMTC (S-methyl-L-thiocitrulline; M5171) was applied and 1400 W (1 μM, W4262) was used as a specific iNOS blocker.

### Tissue lysis, SDS-PAGE, and western blot analysis

Control tissue from the mouse diaphragm or myooids was frozen in liquid nitrogen and mechanically broken up into small pieces in the presence of liquid nitrogen. Cell and tissue whole protein content was extracted with whole-cell lysis buffer containing 20 mM Tris-HCl, pH 7.5, 150 mM NaCl, 1 mM EDTA, 1 mM EGTA, 1% Triton, 2.5 mM Na_4_P_2_O_7_, 1 mM Na_3_VO_4_. Freshly added 1 mM PMSF, 1 mM DTT, 4 µL/ml Protease Inhibitor Cocktail, 10 µL/ml Phosphate Inhibitor, and 150 µL 1 M NaOH. After centrifugation (5 min, 10000 rpm), the protein concentration was determined in the supernatants using a NanoDrop (NanoDrop One, Thermo Scientific) against whole-cell lysis buffer. Diaphragm and myooid protein extracts were separated on pre-cast 4–12% SDS-PAGE NuPAGE® Novex® Bis-Tris Gels gradient gels where 50 µg of total protein was loaded per lane. After electrophoretic separation, proteins were transferred to the PVDF membrane with a 0.45 µM pore size (Amersham Hybond *via* Sigma-Aldrich) using a wet blotting system for higher molecular weight proteins. The membranes were then blocked in TBS-T with 5% non-fat dry milk (Roth GmbH & Co. KG) for 60 min, followed by incubation with the respective primary antibodies in TBS-T with 5% milk at 4°C overnight. All antibodies were purchased from Abcam; primary antibodies and the respective dilution used were against dystrophin (1:100; ab15277), nNOS (1:1000, ab5586), eNOS (1:1000, ab50260), iNOS (1:400, ab15323), SERCA1 (1:1000; ab129104), DHPR (1:500; ab2864), RyR1 (1:500; ab2827), GAPDH (1:10.000; ab181603). Membranes were washed and incubated in TBS-T with 5% milk and secondary antibodies for 1 hour at room temperature. According to the source of primary antibodies, the following secondary HPR-coupled antibodies were used: goat anti-mouse IgG1 (1:50,000; ab97240), goat anti-mouse IgG2a (1:15,000; ab97245) or goat anti-rabbit IgG (1:20,000; ab205718). Membrane strips were washed again and HPR activity was visualized with a chemiluminescence substrate (AceGlow, Peqlab *via* VWR International GmbH) and recorded with an intensified CCD camera. The full membrane length is displayed in Supplementary Figure S1.

### Data analysis

Data recording and analysis were performed using the software LabChart8; graphical representation and statistical analysis were done in GraphPad Prism V.7.0 (GraphPad Software, San Diego, California, United States). Statistically significant outliers were detected by the free online available Outlier Calculator from GraphPad QuickCalcs (*α* = 0.05). Data were tested unpaired using One-way ANOVA with a significance threshold set at 5% (*p* < 0.05). Treated data were compared to control data by a Bonferroni post-hoc statistical test after ANOVA significant results. Data are represented in mean ± SD and the n represents the number of myooid or mice.

## Results

### Formation and characterization of the myooid

In comparison to previously published protocols, additional steps in our protocol simplified and increased the yield of myooids production (Figure 1A). The main differences from previous protocols were seeding the C2C12 cells on a gelatin-covered plastic and carving the PDMS, forming a well of 1.0 cm wide by 1.5 cm long, resulting in an enclosed volume where the cells suffered no hydric stress by accidental removal of all of the media. These procedures also increased myoblast fusion, the number of myooids attached to the sutures, and protected the myooids from mechanical stress, such as fluid movement during media exchanges and from adding or removing the stimulating electrodes. After a period of growth until confluency and differentiation, the average time of the 2-D layers detaching from the plastic surface and forming myooids was between 7- and 21 days (Figure 1B). Once myooids were formed (Figure 1C), they were electrically stimulated for 7 days until the experiment (Figure 1A). All myooids used and analyzed in this study were stimulated and recorded before the 40th day of total culture time. In an independent experiment, several myooids were cultured for up to 6 months without any difference in force production compared with the myooids described here. The CSA value obtained from each myooid was then used to calculate the respective specific twitch and tetanic forces. For CSA, the 180 myooids recorded and analyzed were approximately 1.4 cm long, with a median CSA of 2.36 mm^2^. The measured CSA from matured myooids was consistent across several experiments, suggesting a reliable myooid formation independent of the investigator (Figure 1D). In comparison to the chosen skeletal muscle diaphragm, the myooid expressed all key EC-coupling proteins, including DHPR, RyR, and SERCA (Figure 1E), as well as nNOS and iNOS (Figure 1F). However, eNOS expression was not detected in the analyzed samples (Figure 1F). The full length of each membrane is displayed in Supplementary Figure S1.

### Single twitch and tetanic force production of the myooid

**FIGURE 2:**
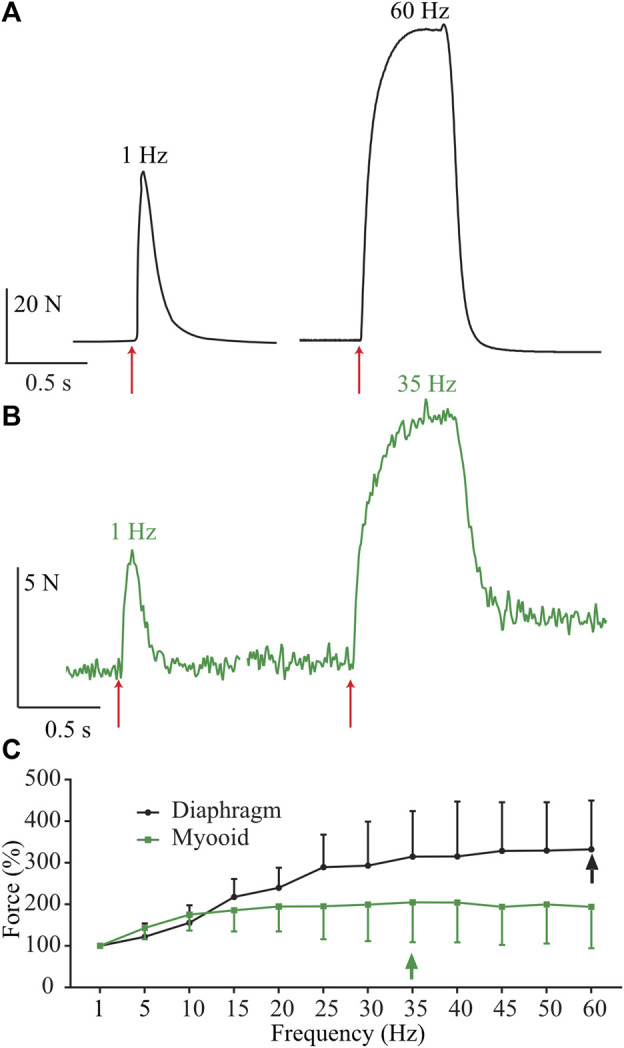
Representative traces of force production from a diaphragm strip and myooids in response to electrical stimulation. The red arrows indicate the moment of 50 V intensity and 20 ms duration for single twitch [left side of panels **(A,B)**], or during 500 ms at 60 Hz for diaphragm strips **(A)** and 35 Hz for myooids **(B)**. **(C)** Summary analysis of the percentage of force-frequency protocol based on single twitch from six diaphragm strips (black line) and twenty myooids (green line). The arrows indicate the maximum force produced during the force-frequency protocol of up to 60 Hz of stimulation frequency.

To evaluate myooid functionality (*n* = 20), we compared them to diaphragm strip specific force harvested from 6-month-old wild-type C57Bl/10 male mice (*n* = 6), using a force-frequency protocol (50V, 5 ms stimulation during 500 ms every 2 min from 1 up to 60 Hz with 5 Hz step increments). To maintain the comparison, only the range from 1 to 60 Hz of stimulating frequency was shown here, even knowing that the stimulating frequency in the diaphragm’s force-frequency relationship is normally higher and up to 200 Hz (Moorwood et al., 2013; Hakim et al., 2019). As expected, raw twitch tetanic force production from a representative mouse diaphragm strip (Figure 2A, black) recorded at the maximum of the force-frequency protocol was greater than recorded in the myooids (Figure 2B, green). The force-frequency relationship normalized to a single twitch (1 Hz) showed that myooids produced their maximum force at 35 Hz while the diaphragm was at 60 Hz (Figure 2C, Supplementary Table S1).

### NO modulates force production in the diaphragm and the myooid

**FIGURE 3:**
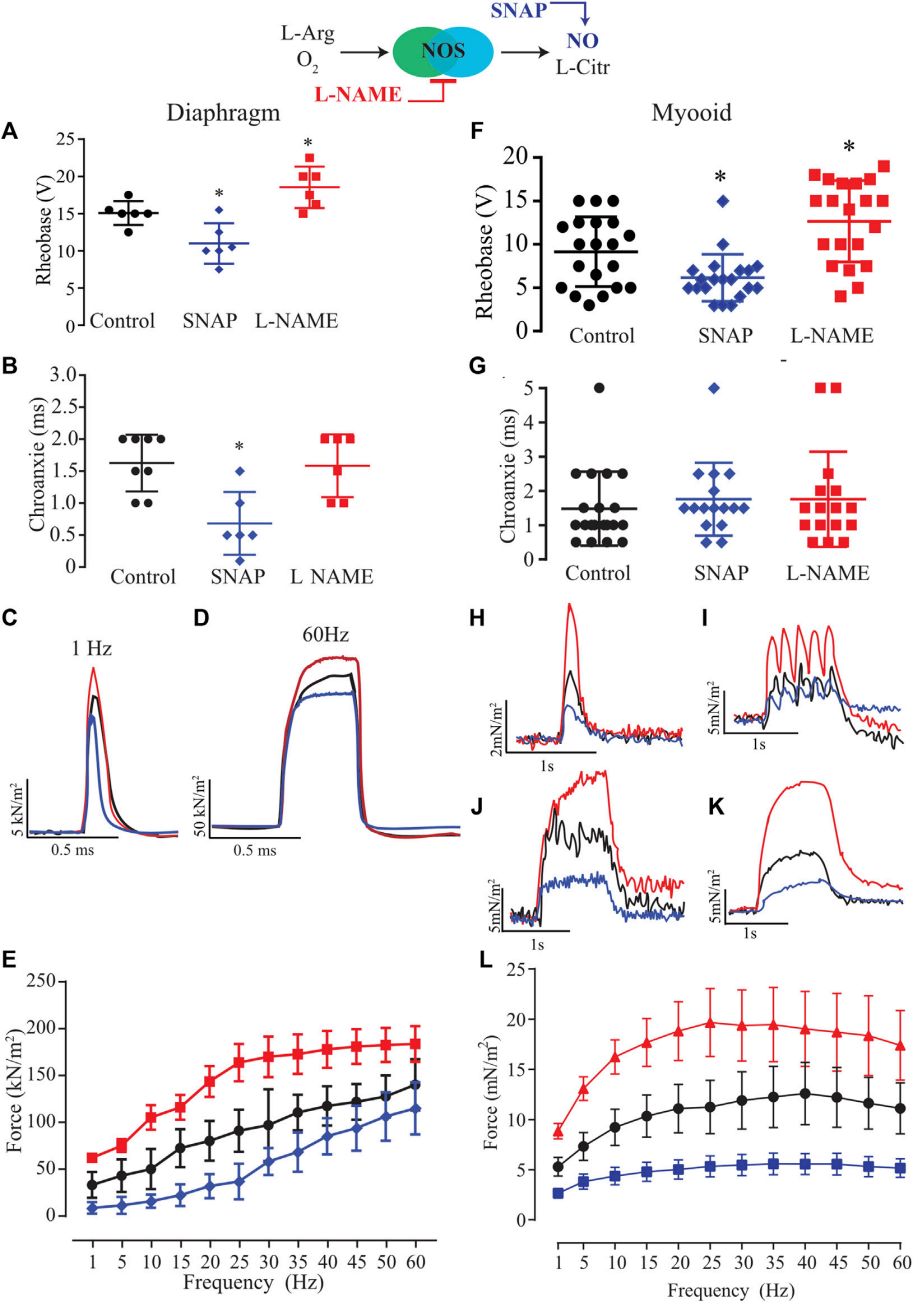
Summary analysis of the effect of exogenous NO and blocking endogenous production on excitability and force-frequency protocol for six diaphragm strips **(A–E)** and twenty myooids per condition **(F–L)**. The response under the effect of 100 µM of SNAP is represented by the blue lines and 5 mM of L-NAME by the red color. **(A,F)** Rheobase response corresponds to the minimal voltage at 100 ms of stimulation for six diaphragm strips per condition. **(B,G)** Chronaxie response corresponds to minimal stimulation duration at double of rheobase for six diaphragm strips and twenty myooids. **(C,D,H,I,J,K)** Representative traces of force production in response to electrical stimulation (50 V, 20 ms) under the effects of SNAP (blue) and L-NAME (red) recorded from diaphragm strips **(C,D)** and myooids **(H–K)**. **(C,H)** represent the response to 1 Hz of stimulation, **(I)** for 5 Hz, J for 20 Hz, K for 35 Hz, and **(D)** for 60 Hz. **(E,L)** summarize the force production in response to increments in frequency of stimulation, from 1 up to 60 Hz for diaphragm strips and myooids, respectively. Statistical comparison is against the control (black) and * represents the threshold of significance set at less than 5%.

Next, we characterized the role of NO on excitability parameters (rheobase and chronaxie) and force production in diaphragm strips from six different male mice using established pharmacological tools such as SNAP (100 μM; blue) as the exogenous NO donor and L-NAME (5 mM; red) as an unspecific NOS blocker. In comparison to the control solution, the exogenous NO or the unspecific NOS blocker significantly altered the rheobase parameter in opposite directions; SNAP decreased it while L-NAME increased it (Figure 3A, Supplementary Table S2). The treatment with SNAP but not L-NAME significantly reduced the chronaxie parameter (Figure 3B). In comparison to the control solution, the force-frequency relationship analysis indicated a significant treatment effect (F_2,15_ = 22.43; *p* < 0.001) as seen in the representative traces of 1 and 60 Hz of stimulation (Figures 3C,D respectively); that in diaphragm strips, SNAP reduced force production, while L-NAME increased it (Figure 3E, Supplementary Table S3). Next, we treated twenty myooids with each pharmacological condition in analogy to the mouse diaphragm to evaluate the same parameters. The rheobase parameter measured from the myooids showed a similar response to the diaphragm, where SNAP significantly reduced it while L-NAME increased it (Figure 3F; Supplementary Table S4). In contrast to the results obtained from the diaphragm strips, these differences were absent once the chronaxie parameters were analyzed (Figure 3G). Next, the effect of NO on force production was evaluated as a function of the frequency of given electrical stimulation. The representative traces depict the responses to stimulations at 1, 5, 20, and 35 Hz (Figures 3H–K), showing a similar pattern of response to electrical stimulation as observed for diaphragm strips. Force-frequency analysis showed a significant effect of both treatments in comparison to the control solution (F_2,57_ = 10,29; *p* < 0.001); thus, unspecific inhibition of NOS significantly increased force production, while and in contrast to L-NAME, SNAP as an exogenous NO donor reduced force production (Figure 3L; Supplementary Table S5). These results suggest that NO modulated the myooid force production in a similar manner to that observed in diaphragm strips.

### NO similarly altered biophysical parameters in both diaphragm and myooid tissues

**FIGURE 4:**
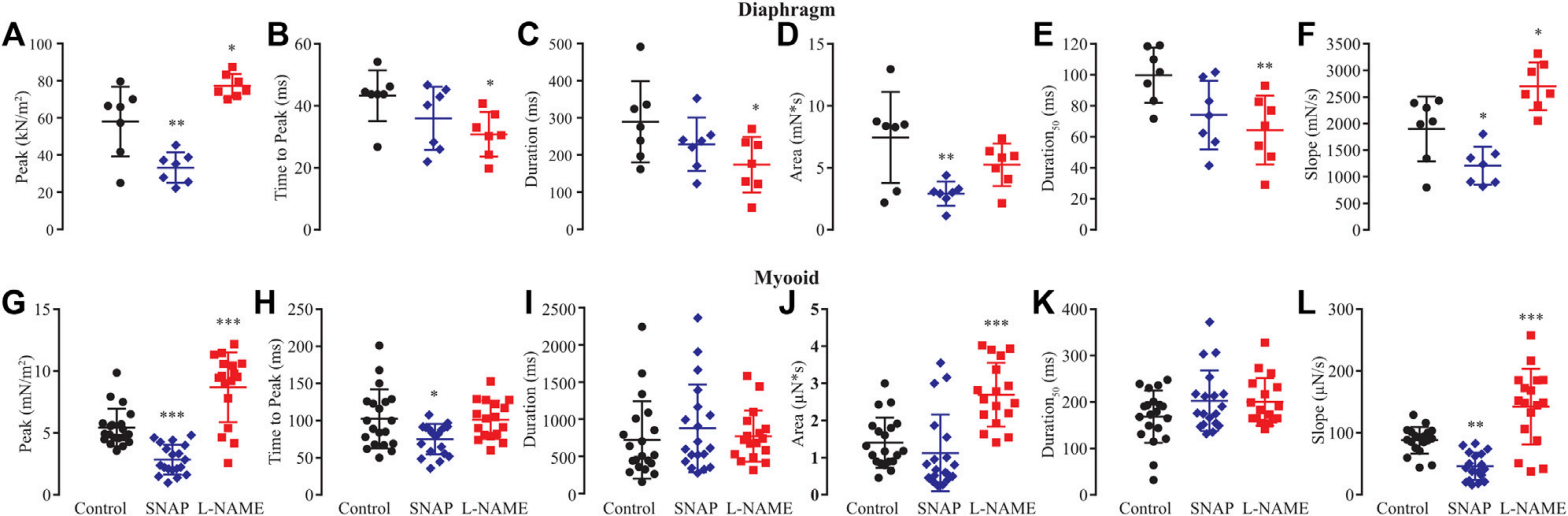
Summary of the effect of SNAP (red) and L-NAME (blue) on biophysical parameters obtained from six diaphragm strips and twenty myooids after a single-twitch stimulation. The control data are represented in black. Panels **(A)** through **(F)** represent the six biophysical parameters analyzed for diaphragm strips, while **(G–L)** is the summary analysis for the myooids. The parameters analyzed were peak **(A,G)**, time to peak **(B,H)**, duration of 95% of the response **(C,I)**, area under the curve of the response **(D,J)**, duration at 50% of the peak **(E,K)** and slope of the rising force production after the electrical stimulation **(F,L)**. The symbols *, **, and *** represent the significant thresholds below 5%, 1%, and 0.1%, respectively.

Based on previous results showing that NO modified rheobase, chronaxie, and single twitch stimulation, we next analyzed six biophysical parameters produced by 1 Hz (single twitch) of electrical stimulation regarding whether NO would interfere with peak, time to peak, duration, area, duration at 50%, and slope of contraction in both the diaphragm and myooids (Figure 4, Supplementary Table S6). In diaphragm strips, SNAP significantly reduced peak (Figure 4A), area (Figure 4B), and slope (Figure 4F), while L-NAME significantly reduced time to peak (Figure 4B), duration (Figure 4C), duration_50_ (Figure 4E), and significantly increasing the peak (Figure 4A) and slope (Figure 4F) parameters. Similar to diaphragm strips, the administration of SNAP to myooids significantly reduced the peak (Figure 4G), time to peak (Figure 4H), and slope (Figure 4L) parameters, while unspecific blockage of NOS with L-NAME significantly increased the peak (Figure 4G), area (Figure 4J), and slope (Figure 4L). These results support a similar effect of NO modulation of force production in myooid as in diaphragm strips. Therefore, we used myooids as a valid model of skeletal muscle to evaluate the effect of NO on force production in all further experiments.

### NO signaling pathway was involved in the modulation of force production in myooids

**FIGURE 5:**
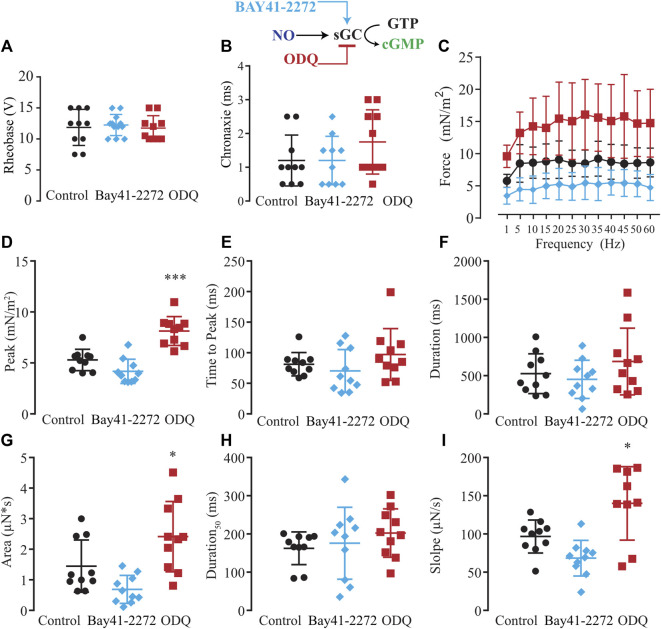
Summary of the effect of Bay41-2272 and ODQ on sGC’s role in myooid response to electrical stimulation. Myooids perfused with control solution are represented by the black color, light blue for myooids treated with sGC activator Bay41-2272, and dark red for those treated with sGC blocker ODQ. **(A)** Rheobase and **(B)** Chronaxie summarized the excitability of the myooids under subthreshold stimulation. **(C)** Force frequency protocol. **(D–I)** analyses of the biophysical parameters in response to single-twitch stimulation. Each condition contained ten myooids and the symbols * and *** represent the significant thresholds below 5% and 0.1%, respectively.

Considering the two possible NO signaling pathways, NO-sGC-PKG and post-translational modifications produced by ONOO^−^ such as S-nitrosylation and nitration, we evaluated which NO signaling pathway would be responsible for the modulatory effects on excitability and force production. First, we tested the hypothesis of whether the activation of sGC with Bay-412272 (10 µM) would reduce force production as seen with SNAP or whether sGC blockage with ODQ (10 µM) would replicate the effect observed from blocking endogenous NO synthesis with L-NAME and thus enhance force production. In comparison to ten control myooids, both Bay 41-2272 (*n* = 10) and ODQ (*n* = 10) did not differ significantly on the rheobase (Figure 5A) and chronaxie (Figure 5B) parameters (Supplementary Table S7). The force-frequency protocol showed that Bay 41-2272 reduced force production similarly to SNAP, while ODQ increased force production, as was observed with L-NAME (Figure 5C; Supplementary Table S8). These results suggest that the pharmacological blockage of sGC leads to the force response as observed with SNAP and L-NAME, respectively. Analyses of the biophysical parameters suggested a similar response as observed with SNAP and L-NAME, where only ODQ significantly increased the peak, the area, and slope (Figures 5D–I; Supplementary Table S9). No significant differences were observed with Bay 41-2272, nor other biophysical parameters under ODQ treatment.

**FIGURE 6:**
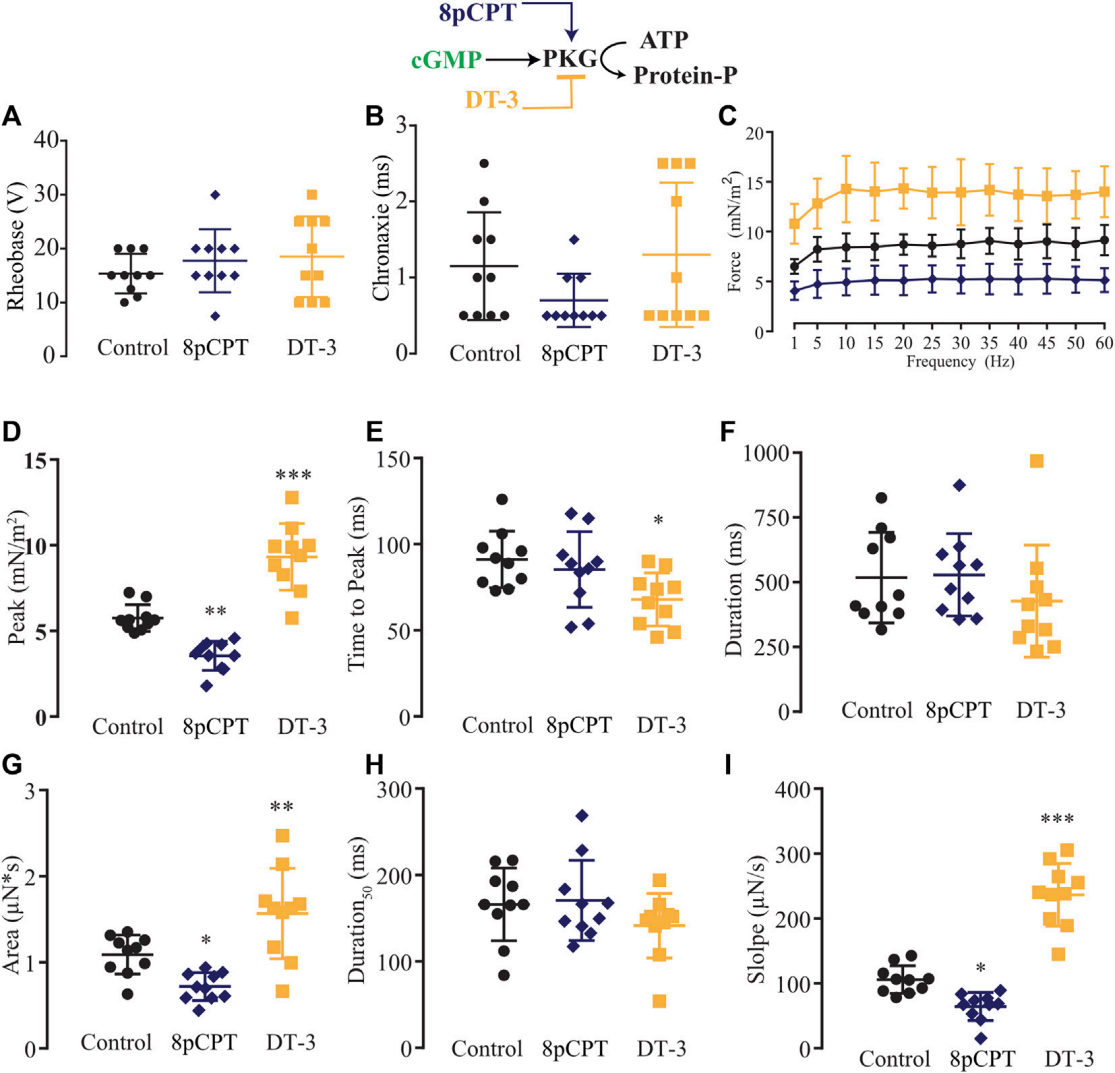
Summary of the effect of 8-pCPT and DT-3 on PKG role in myooid response to electrical stimulation. Myooids perfused with control solution are represented by the black color, dark blue for myooids treated with PKG activator 8-pCPT, and gold color for those treated with PKG blocker DT-3. **(A)** Rheobase and **(B)** Chronaxie summarized the excitability of the myooids under subthreshold stimulation. **(C)** Force frequency protocol. **(D–I)** analyses of the biophysical parameters in response to single-twitch stimulation. Each condition contained ten myooids and the symbols *, **, and *** represent the significant thresholds below 5%, 1%, and 0.1%, respectively.

Next, we modulated pharmacologically one step downstream of the NO signaling pathway, activating or blocking PKG with 8pCPT (10 µM) or DT-3 (100 nM), respectively (Figure 6). In comparison to the control solution (*n* = 10), no alteration in rheobase and chronaxie parameters was observed under the treatment of 8pCPT (*n* = 10) nor DT-3 (*n* = 10; Figures 6A,B; Supplementary Table S10). The force-frequency relationship was changed similarly as observed before with the pair of agonist and antagonist such as SNAP and L-NAME, and Bay 41-2272 and ODQ, in which the former activates the NO-sGC signaling pathway, reducing force production while the latter blocks it, having the opposite significant effect (Figure 6C; Supplementary Table S11). The effect of the pharmacological tools over PKG was more pronounced than it was in the previous experiments, where activating PKG with 8pCPT significantly decreased the peak, area, and slope, while blocking it with DT-3 significantly increased the peak, time to peak, area, and slope (Figures 6D–I; Supplementary Table S12).

**FIGURE 7:**
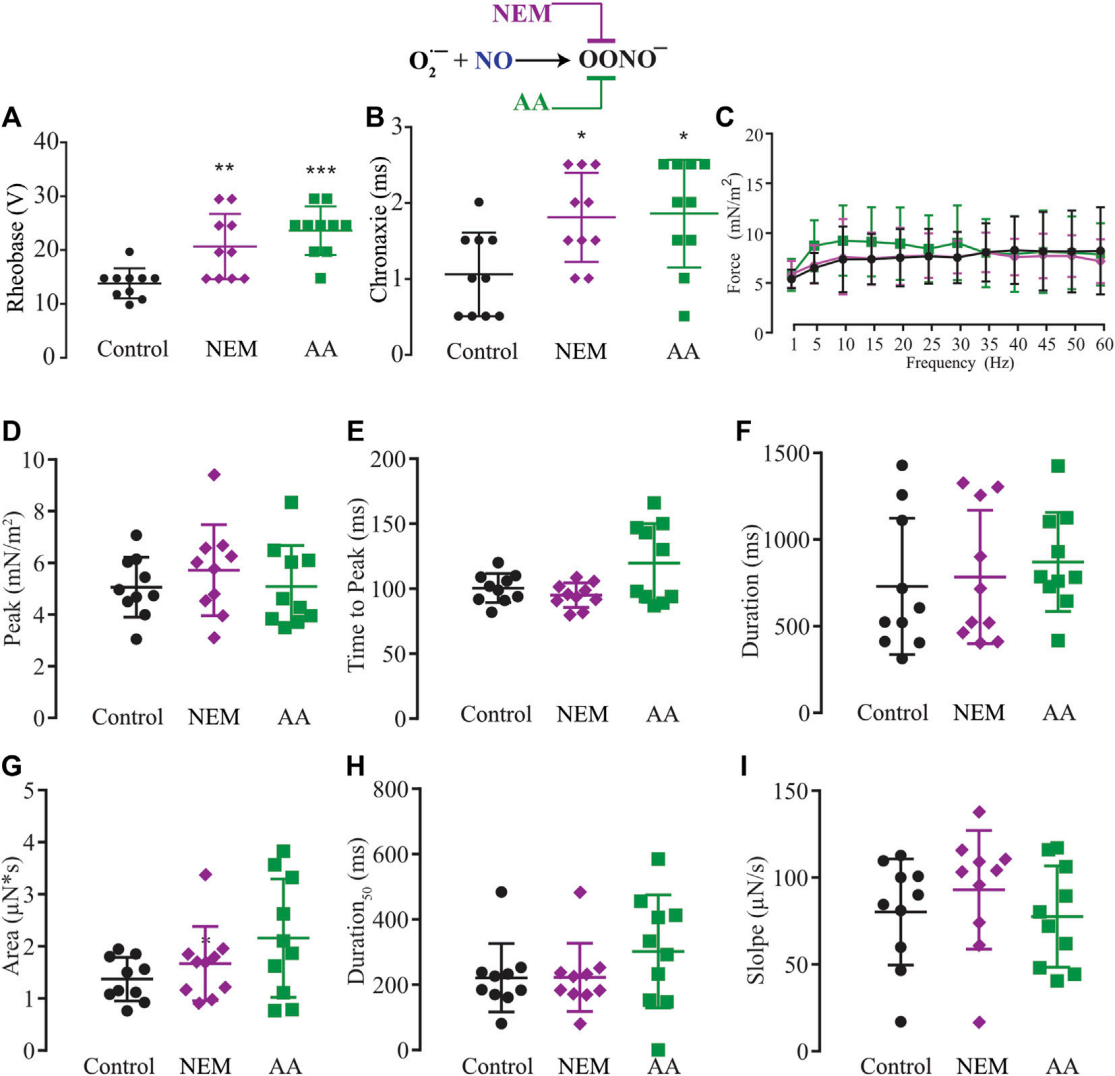
Summary of the effect of NEM and AA on the function of S-nitrosylation in myooid response to electrical stimulation. Myooids perfused with control solution are represented by the black color, violet for myooids treated with NEM, and green for those treated with AA. **(A)** rheobase and **(B)** chronaxie summarized the excitability of the myooids under subthreshold stimulation. **(C)**. Force frequency protocol. **(D–I)** analyses of the biophysical parameters in response to single-twitch stimulation. Each condition contained ten myooids and the symbols *, **, and *** represent the significant thresholds below 5%, 1%, and 0.1%, respectively.

The NO has two different signaling pathways, and NO-sGC-PKG showed a modulatory effect on force production. However, we also evaluated the possibility of an effect *via* the other signaling pathway, the post-tranlational modifications *via* ONOO^−^ (S-nitrosylation or nitration). Since the exogenous application of superoxide to transform the NO into peroxynitrite might result in a cytotoxic effect, we decided to use N-ethylmaleide (NEM) and ascorbic acid (AA) to block endogenous peroxynitrite formation of S-Nitrosylation (Aracena-Parks et al., 2006; Su et al., 2013; Dutka et al., 2017; Ferrer-Sueta et al., 2018) or nitration (Viner et al., 1999; Squier and Bigelow, 2000; Uda et al., 2020). Ten myooids under the effect of NEM (100 μM) or ten others treated with AA (1 mM) showed significant increases in both rheobase and chronaxie in comparison to the control (Figures 7A,B; Supplementary Table S13). The force-frequency and the other six single-twitch parameters measured did not show differences in myooids superfused with control solution (Figures 7C–I; Supplementary Tables S14, S15). Together, these results suggest that force production is mediated by the sGC-PKG signaling pathway while the sensitivity response to electrical stimulation is *via* S-nitrosylation or nitration produced by peroxynitrite.

### nNOS- and iNOS-derived NO modulated different aspects of myooid EC coupling

**FIGURE 8:**
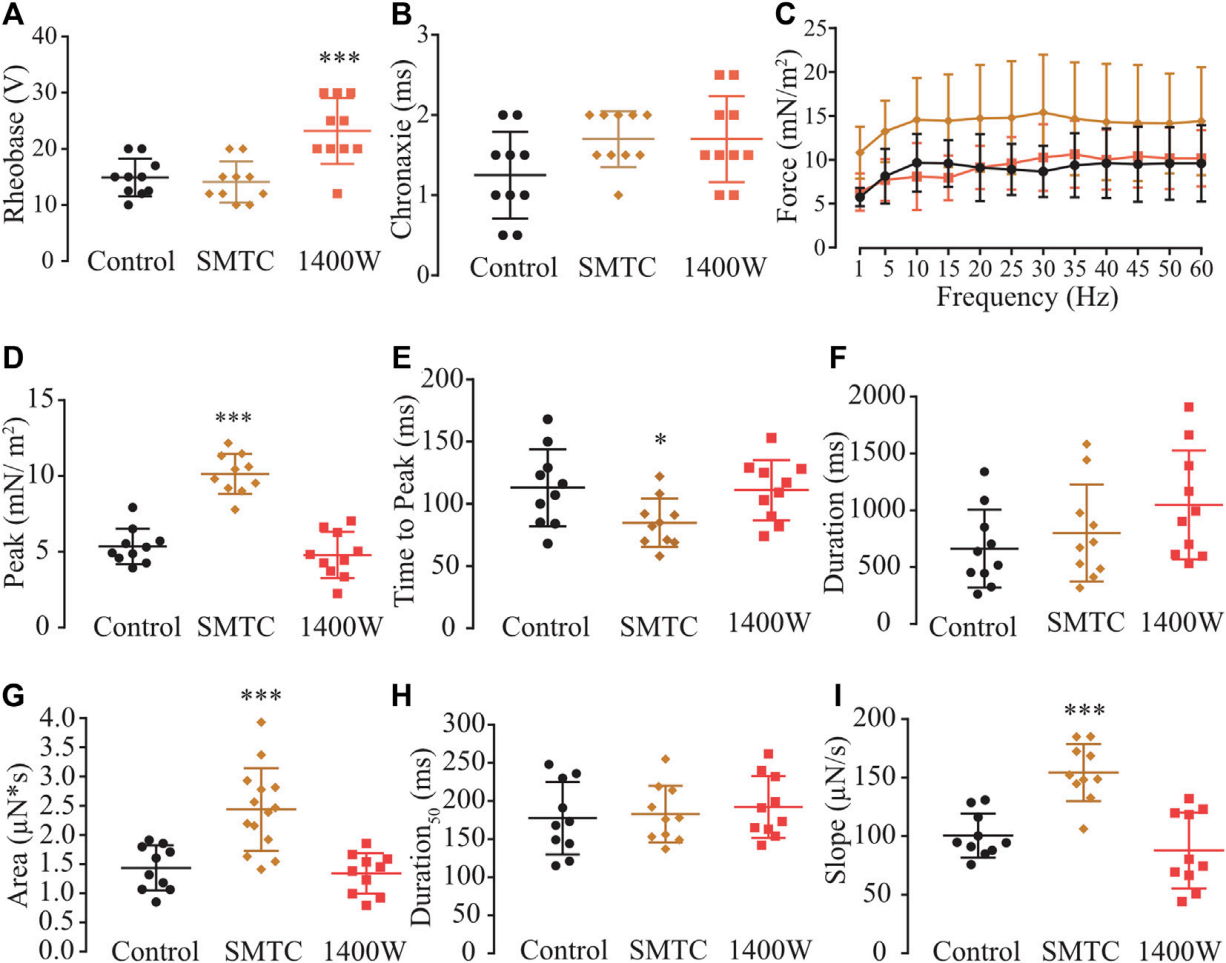
Determination of the function of each NOS isoform expressed in the myooid in response to electrical stimulation. Myooids perfused with control solution are represented by the black color, copper for myooids treated with nNOS blocker SMTC, and coral red for those treated with iNOS blocker 1400 W. **(A)**. Rheobase and **(B)**. Chronaxie summarized the excitability of the myooids under subthreshold stimulation. **(C)**. Force frequency protocol. **(D–I)** analyses of the biophysical parameters in response to single-twitch stimulation. Each condition contained ten myooids and the symbols *, **, and *** represent the significant threshold below 5%, 1%, and 0.1%, respectively.

Biochemical evidence showed that myooids expressed nNOS and iNOS isoforms (Figure 1D), while physiological evidence showed that the rheobase parameter was modified with SNAP or L-NAME on both the diaphragm (Figure 3A) and myooids (Figure 3F), as well as with NEM and AA. However, the rheobase was insensitive to agonists or antagonists of the NO-sGC-PKG signaling pathway, while the force production was altered by the NO-sGC-PKG signaling pathway but not in the presence of peroxynitrite blockers. Next, we evaluated whether either or both expressed isoforms would modulate the sensitivity to subthreshold electrical stimulation and/or contraction force production. To address this question, ten myooids were incubated with specific blockers for nNOS (SMTC, 100 nM) or for iNOS (1400W, 1 μM). Only the rheobase parameter for the myooids treated with SMTC (blocking endogenous nNOS activity and leaving the iNOS able to produce NO) was significantly higher in comparison to the untreated control (Figure 8A; Supplementary Table S16). This effect on rheobase was not observed when iNOS activity was blocked with 1400 W and the nNOS was able to produce NO. Similar to the observation in Figure 3, no difference in chronaxie parameters was observed with either SMTC or 1400 W (Figure 8B; Supplementary Table S16). The force-frequency response and the single-twitch parameters were significantly different from the control solution under the effect of 1400W, however, no effect was observed with SMTC treatment (Figure 8C; Supplementary Table S17). In the same line, only treatment with 1400 W significantly altered twitch biophysical parameters, while SMTC caused no significant effect (Figures 8D–I; Supplementary Table S18). These results suggest a differential role in muscle contraction where nNOS would produce NO to reduce force production while iNOS-derived NO would increase the sensitivity of myooids in response to sub-threshold electrical stimulation.

## Discussion

Here, we presented a new and simplified method for tissue engineering of muscle fibers using the C2C12 cell line, previously termed as myooids (Dennis et al., 2001). This method further optimized and developed the original idea (Vandenburgh et al., 1996; Dennis et al., 2001; Kosnik et al., 2001; Fujita et al., 2009; van der Schaft et al., 2013), allowing us to elucidate the modulatory role of NO, its signaling pathways, and the sources of muscle contraction in the myooids. Based on nNOS and iNOS protein expression observed in the myooid and supported by a previous report demonstrating that eNOS is not expressed in C2C12 cells (He et al., 2003), we analyzed only the function of nNOS and iNOS. As previously shown (Kobzik et al., 1994; Eu et al., 2003) and thus hypothesized here, nNOS-derived NO negatively modulates force production in both the diaphragm and myooid muscles; however, interestingly, we found that iNOS-derived NO was responsible for modulating the sensitivity of myooid response to sub-threshold electrical stimulations. The initial comparison between the diaphragm and myooids showed similarities in force production in response to electrical stimulation and the modulatory effects of NO, supporting the relevance of the technique for answering biological questions that otherwise would require a large number of animals.

### Protocol improvements resulted in efficient tissue engineering of muscle fiber from C2C12 cells

The original idea of constructing a muscle fiber from either primary cells or an established cell-line was proposed by Vandenburg (Vandenburgh et al., 1991a; Vandenburgh et al., 1991b; Vandenburgh et al., 1991c; Vandenburgh et al., 1996) and then further developed by Dennis and Kosnik (2000), (Dennis et al., 2001; Kosnik et al., 2001). This simplified model of a skeletal muscle fiber technique opened a new research field for the exploration of different techniques of development and thus offers the ability to answer questions that otherwise would require a large number of animals. Based on the progress from Dennis and Kosnik (2000), Kosnik et al. (2001), and Fujita et al. (2009), we made further optimization to obtain a simpler and faster method for building a 3-D tissue engineering skeletal muscle fiber, also referred to as a myooid. One of the key aspects of the developed technique was the observation of classical muscle structures in 3-D tissue engineering from primary cell culture and from the C2C12 cell line, such as sarcomeres, t-tubules, sarcoplasmic reticulum, and the presence of mitochondria (Parton et al., 1997; Carozzi et al., 2000; Borschel et al., 2004; Park et al., 2008; Fujita et al., 2009; Khodabukus and Baar, 2009; Fujita et al., 2010; Fugier et al., 2011; Martin et al., 2015; Orfanos et al., 2016; Martin et al., 2017).

The current method is schematized in Figure 1, and shows that a simple adaptation for preparing the plates, such as carving the PDMS out and then cutting in the middle of each short-axis, thus holding the frayed sutures close enough to the C2C12 cells for them to be grasped and used as a tendon, saved time in preparation of the plates and increased yoid yield. Since the growth and further differentiation of C2C12 cells from myoblasts into myofiber were not affected by the plastic surface of the 6-well plate, it was not necessary to use any special coating, but the inclusion of gelatin for cell culture improved the yield. Within less than 30 days from seeding (day 0), myooids were ready for electrical stimulation and, once formed, we could not detect any significant differences in aspect and function compared to plates cultured for 6 months.

### Force response and effect of NO on myooid vs. murine diaphragm muscle

Our data supported previous observations showing that myooids produce lower raw and normalized force in comparison to a muscle sample from an adult animal (Dennis and Kosnik, 2000), demonstrating that most of its mass is composed of non-contractile machinery. Nevertheless, the capacity of myooids to respond to electrical stimulation to generate force makes them a useful *in vitro* model for muscle physiology, genetic or biochemical approaches. Moreover, a detailed analysis performed here showed important differences, such as the maximal single twitch (5 N in the myooid vs. 20 N in the diaphragm) and tetanic force (35 Hz in the myooid vs. 60 Hz in the diaphragm). It is relevant to clarify that our force-frequency protocol was designed to compare the force production between myooids and the diaphragm even though it is well known that the maximal stimulation frequency for the diaphragm muscle strip is as high as 200 Hz (Moorwood et al., 2013; Hakim et al., 2019). In the control solution, the sensitivity response to electrical stimulation (measured through the rheobase and chronaxie) did not differ between both diaphragm and myooid models. The rheobase and chronaxie parameters from our myooid differed from a previous report (Dennis et al., 2001), which could be explained by differences in the process of engineering the myooid, the protocol of stimulation for both maturation and response, and equipment. The major functional difference between the myooid and the diaphragm was observed on the scale of single twitch analyses, which can be explained by the development and maturity of the muscle, a known issue that must be improved in tissue engineering technique. Nevertheless, the similarity of response between myooids and the diaphragm supports the idea that myooids are a valuable skeletal muscle tool for new pharmacological and genetic approaches. We used this concept to study the effect of NO on myooids and compared the response to the diaphragm. Interestingly, exogenous NO released by SNAP induced a significant reduction of rheobase in both tissues, and the opposite effect was observed in the presence of L-NAME. These effects were absent in the chronaxie analysis, suggesting a differential effect of NO on sub-threshold electrical stimulation. It is important to point out here a relevant aspect of the experimental design. One is the use of carbogen in the perfusion solution and the consequence of high pO_2_ in the muscle response. It was previously shown that high pO_2_ (20%) reduces single twitch and tetanic forces and even reverses the effect of NO on force production (Eu et al., 2000; Eu et al., 2003). Although it is a key aspect of muscle metabolism during activity that must be considered in the future, here we decided to use carbogen instead of air to bubble the solutions to avoid anoxia in the core of the myooid and to use a comparable published composition of the perfused solutions. The force-frequency protocol revealed that SNAP reduced the force production while L-NAME had the opposite effect on both tissues. These differential responses of both pharmacological tools have already been reported in different muscles (Kobzik et al., 1994; Andrade et al., 1998; Reid, 1998; Xiyuan et al., 2017), but never in tissue engineered muscles.

We also evaluated the NO effect on six biophysical parameters obtained from single-twitch response measured on both the diaphragm and myooids. Due to the already mentioned differences in maturation of these two muscle tissues, only peak (as maximal force production) and slope (as the rate of force being produced) matched, suggesting that NO would modulate twitch force production in myooids in the same way as in the diaphragm strips. Therefore, the differences between the diaphragm and myooids regarding force production and biophysical parameters analyzed here could be owing to a combination of several reasons, such as maturity of the tissue, the size of the tissue, and the number of samples used. There are, nevertheless, strategies to reduce these differences, such as adding different growth factors in the differentiation medium, seeding surface, serum, electrical field stimulation, decellularized scaffolds, or co-culture with fibroblasts or neuronal cells (Fuchs et al., 2003; Borschel et al., 2004; Cooper et al., 2004; Fujita et al., 2009; Khodabukus and Baar, 2009, 2014; Khodabukus et al., 2015; Martin et al., 2017; Ostrovidov et al., 2017; Shima et al., 2020; Gaffney et al., 2021). Despite this great progress, some adjustments are still needed before establishing a universal protocol to collect data that is more consistent and comparable with animal-sourced muscle fiber.

The expression of different NOS isoforms has been extensively reported in different skeletal muscles, demonstrating and further confirming the expression of all three isoforms (Gath et al., 1996; Thompson et al., 1996; Reid, 1998; Stamler and Meissner, 2001; Mosqueira et al., 2013; Xiyuan et al., 2017). nNOS is reportedly expressed close to the sarcolemma in the diaphragm, EDL, and soleus muscles (Reid, 1998; Hirschfield et al., 2000), and is associated with dystrophin *via* syntrophin through the PDZ domain (Mosqueira et al., 2013). iNOs expression has been reported in the sarcolemma associated with caveolin-3 of the gastrocnemius and diaphragm muscles (Gath et al., 1996; Thompson et al., 1996). Finally, eNOS has also been reported in muscle using immunohistochemistry and western blotting with a stronger signal on the vessels (Fujii et al., 1998; Reid, 1998). In addition, the effect of NO on force production was also evaluated, indicating that unspecific blockage of all three isoforms induced an increase in force production, as well as pharmacological blockage with nNOS specific blockers, such as 7-nitroimidazole and SMTC (Reid, 1998). The production of NO in eNOS-KO mice is significantly higher during muscle contraction in comparison to the resting period, but no effect was observed among wild-type and eNOS-KO mice (Hirschfield et al., 2000), supporting other evidence that eNOS-derived NO does not participate in the modulatory role of force production. It has been previously demonstrated that C2C12 cells do not express the eNOS isoform (Hirschfield et al., 2000; He et al., 2003), which was confirmed within myooids. Therefore, the use of myooids as a muscle model simplified the understanding of the role of NO and its source in skeletal muscle function by expressing two (nNOs and iNOS) isoforms. As also observed here, different NO donors decrease force production, supporting evidence that NO acts as a negative modulator of force production (Kobzik et al., 1994; Gath et al., 1996; Reid, 1998); and contrarily, blocking NOS activity *via* gene knockout or pharmacological treatment increases force production (Kobzik et al., 1994; Reid, 1998; Hirschfield et al., 2000). However, there are some open questions regarding the role of each isoform during EC-coupling and which signaling pathway would be used to modulate force production. The effect of NO on the diaphragm in response to electrical stimulation was used to compare to the one obtained from the myooid, and as observed in the diaphragm, the NO donor SNAP reduced the force production recorded in the force-frequency protocol, while treatment with the unspecific blocker L-NAME increased force production. Therefore, our data support these previous data and extend them to the myooid, showing that a similar response obtained in mature skeletal muscle is also observed in the tissue-engineered muscle fiber using the C2C12 cell line.

### Differential signaling pathways to modulate force production

Our technique was developed to produce tissue-engineered muscle fibers from the C2C12 cell line on a large scale and in a shorter time span. Consequently, we were able to analyze ten myooids per condition, thus improving statistical analysis and the biological meaning of data compared to existing experiments with mice. We decided to use pharmacological tools contrary to a genetic approach owing to the possibility of compensatory responses from the expressing isoform, as it has been described in vascular smooth muscle cells, cardiomyocytes, and skeletal muscles (Huang et al., 2002; Khan et al., 2003; Talukder et al., 2004; Takaki et al., 2008) (Li et al., 2011; Nogami et al., 2020). Moreover, knocking out nNOS in skeletal muscle alters the expression of other proteins (Ramachandran et al., 2013), increases ROS production (Da Silva-Azevedo et al., 2009), and modifies metabolic pathways (Hong et al., 2016) that might result in a differential EC-coupling response. Additionally, the incubation time between the pharmacological agents used here and the recording of myooid activity was within the gene expression period for an observable change in the protein level, supporting the hypothesis of a differential role for both nNOS and iNOS isoforms during EC-coupling. These aspects mentioned above were relevant owing to the possibility of independent evaluation without interference of two known pathways, through which NO exerts its effect: either *via* NO-sGC-PKG or post-translational modification from peroxynitrite nitrosylating thiol groups of cysteines, well-known as S-nitrosylation (SNO) (Eu et al., 2000; Mosqueira et al., 2013) or *via* nitration of tyrosine rings (Viner et al., 1999; Squier and Bigelow, 2000; Uda et al., 2020). Taking advantage of the standard pharmacological tools, we first used the sGC’s activator Bay41-2272 and the blocker ODQ (Drenning et al., 2008; Makris et al., 2010; Mosqueira et al., 2021). Leaving aside the results of excitability parameters, the results from force-frequency and single twitch biophysical parameters showed that Bay41-2272 and ODQ induced similar effects as observed with SNAP and L-NAME, respectively, suggesting that NO would modulate force production *via* sGC. The next point of investigation addressed PKG, which is activated by cGMP-produced sGC, using a specific agonist [8-pCPT-cGMP Ishikawa et al. (2019), Mosqueira et al. (2021)] and a blocker (DT-3 (Takahashi et al., 2008)). Supporting previous results, the analysis of rheobase differed from SNAP and L-NAME, but no effect was seen in the analyses of rheobase for Bay41-2272 and ODQ. The force-frequency and single twitch parameters showed a similar pattern as observed with SNAP and L-NAME, as well as for Bay41-2272 and ODQ, supporting the hypothesis that NO would modulate force production *via* the NO-sGC-PKG signaling pathway.

Next, we evaluated whether the post-translational modification from peroxynitrite production to form either SNO or nitration would take part in the modulatory effect observed above using two compounds in skeletal muscle (Porter Moore et al., 1999; Salanova et al., 2013; Dutka et al., 2017): N-ethylmaleimide [NEM (Xiyuan et al., 2017; Mosqueira et al., 2021)] and ascorbic acid [AA (Holmes and Williams, 2000; Ferrer-Sueta et al., 2018; Mosqueira et al., 2021)]. Surprisingly, both antioxidants produced the same effect on rheobase, increasing it to the same level as it was observed with L-NAME. Moreover, the force-frequency and the twitch parameters were not affected by the presence of either NEM or AA, suggesting that the action of peroxynitrite is a physiological effect that modulates the sub-threshold excitability of myooids. However, contradicting data on skeletal muscle shows that SNO reduces contractility *via* inhibiting contractility apparatus, myosin, and/-or RyR1 activity (Porter Moore et al., 1999; Nogueira et al., 2009; Dutka et al., 2011; Dutka et al., 2017), where others have reported an increase in RyR1 activity and force production (Eu et al., 2000; Hart and Dulhunty, 2000; Sun et al., 2001; Sun et al., 2003). Although our results do support a positive effect of the NO-ONOO^-^ pathway on the subthreshold stimulation excitability of myooids under normal conditions, which is essential to initialize force production, it is still unknown at what level of EC-coupling this occurs. Furthermore, the differences between those previous reports showing a reduction in force production *via* actions of peroxynitrite and our results lying on the amount and source of NO, where we reported an effect from an endogenous NO production instead of exogenous application of a NO donor. Another relevant point is the duration of the incubation of NO donors (continuously and constant for several minutes) vs. endogenous NO production. We recently observed in isolated ventricular cardiomyocytes that there is a transient NOS-dependent NO production after electrical stimulation (Mosqueira et al., 2021). Here, we did not measure endogenous NO production in myooids, leaving an open question regarding the endogenous NO production after electrical stimulation. Taken together, these results showed the role of the two NO signaling pathways, where the NO-sGC-PKG pathway would negatively modulate force production in response to supra-threshold electrical stimulation, while the NO-ONOO^-^ pathway would increase the excitability to sub-threshold electrical stimulation.

### Two NOS isoforms for two distinct functions

Taking advantage of the protein expression data, we analyzed the same parameters as discussed above using SMTC and 1400 W as specific blockers of nNOS and iNOS, respectively (Garvey et al., 1997; Khan et al., 2003; Barker et al., 2005; Copp et al., 2011; Jin et al., 2013; Mosqueira et al., 2021). The analysis of rheobase showed a similarity between the control and myooids treated with SMTC, where only iNOS was able to produce endogenous NO; however, analysis of the rheobase of myooids treated with 1400W suggested that iNOS-derived NO increased the voltage-dependent excitability but did not affect the duration of the response. Alteration of the force-frequency and twitch parameter responses were absent in the presence of 1400 W but enhanced with SMTC, supporting previous results showing that nNOS-derived NO reduces force production (Kobzik et al., 1994; Li et al., 2011) but not iNOS-derived NO (Nogami et al., 2020). The isoform-dependent NO effect has been extensively described in skeletal muscle (Kobzik et al., 1994; Bellinger et al., 2009; Nogueira et al., 2009) and other tissues, such as the carotid body and cardiomyocytes (Valdes et al., 2003; Mosqueira et al., 2021). The results presented here support previous reports on differential NOS isoform-dependent effects and extend the understanding of how the nNOS-derived NO could negatively modulate supra-threshold muscle contraction, most probably *via* the NO-sGC-PKG signaling pathway, while iNOS-derived NO would enhance the excitability of myooids under sub-threshold responses *via* the actions of peroxynitrite.

### Limitations

The major limitation of myooids as a method to completely substitute animal-derived muscle in research is based on their formation and maturation. A cell line such as C2C12 does not form a muscle fiber as obtained directly from an animal owing to the complex, sequential, and long-term development and growth of the muscle up to adulthood, which is the usual age used in basic and advanced research. To overcome these pitfalls, different media, growth factors, serums, and electrical stimulation have been successfully applied (Fujita et al., 2009; Khodabukus and Baar, 2009; van der Schaft et al., 2013; Khodabukus, 2021). Therefore, comparison to *in vivo* muscle is still needed to identify the limitations of the method and to make improvements in the protocol by shortening the anatomical, biochemical, and physiological distances between them.

An alternative to this method with C2C12 or cell lines is the use of stem cells (Park et al., 2008; Khodabukus et al., 2015), which presents a major advantage over cell lines because they can produce muscle fibers with similar anatomy to *in vivo* muscles. The disadvantage of the stem cell strategy, on the other hand, is the necessity of using animals and the lengthy cell culture process until a workable muscle is obtained (Park et al., 2008; Khodabukus et al., 2015).

Finally, although it is not possible with the current data to determine which proteins are modulated by the NO-sCG-PKG or NO-ONOO^-^ signaling pathways, the current model is suitable for such a large and complex experimental design.

## Conclusion

One of the key advantages of tissue engineering is the ability to develop new platforms to evaluate different strategies in a simpler model, which, in most cases, reduces the number of animals without sacrificing the relevance of the data when compared to the original tissue. Here, we took this advantage by analyzing 180 myooids to evaluate in detail the role, source, and signaling pathway of NO in skeletal muscle contraction. Another key benefit of using the C2C12 cell line is the capacity to make genetic alterations that otherwise would be lethal in an animal. In summary, tissue engineering muscle fibers from the C2C12 cell line allowed us to describe the differential roles of nNOS and iNOS in response to electrical stimulation, suggesting that the nNOS-derived NO could influence total force production while the iNOS-derived NO would modulate the sensitivity of the EC coupling. Further development of this method will allow the identification of alternative treatments for muscle diseases and expand the understanding of muscle physiology.

## Data Availability

The raw data supporting the conclusions of this article will be made available by the authors without undue reservation.
