# Supplementary material for: nNOS-derived NO modulates force production and iNO-derived NO the excitability in C2C12-derived 3D tissue engineering skeletal muscle via different NO signaling pathways
Source: Front Physiol. 2022 Aug 15;13:946682. doi: 10.3389/fphys.2022.946682 (PMC9421439; doi:10.3389/fphys.2022.946682)
Supplement: Supplementary file 1 [file Table1.DOCX]

Supplementary Material

**nNOS-derived NO Modulates Force Production and iNO-derived NO the excitability in C2C12-derived 3-D Tissue Engineering Skeletal Muscle via different NO Signaling Pathways.**

Matias Mosqueira^1*^, Lisa-Mareike Scheid^1,2^, Dirk Ollech^1,3^, Dominik Kiemel^1,4^, Almut Lutge^1,5^, Mona Rheinberger^1,6^, Tim Heißenberg^1,7^, Lena Pfitzer^1,8^, Talisa, Richardt^1,9^, Lisa Engelskircher^1,10^, Umut Yildiz^1,11^, Isabel Porth^1,12^.

*** Correspondence:** Matias@physiologie.uni-heidelberg.de

# Supplementary Figure

**
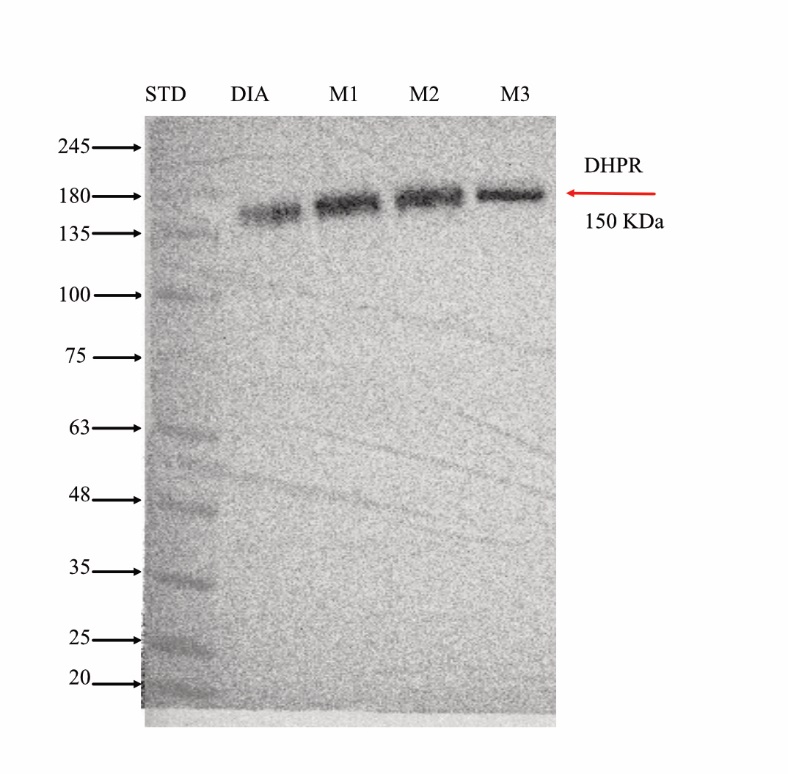
Supplementary Figure 1.** Each immunoblotting image was overlayed with the image of the membrane, obtaining a merged imaged of the bands and the standard of molecular weight. The black arrows inidicate the standard of molecular weight and the red arrow the protein of interest.


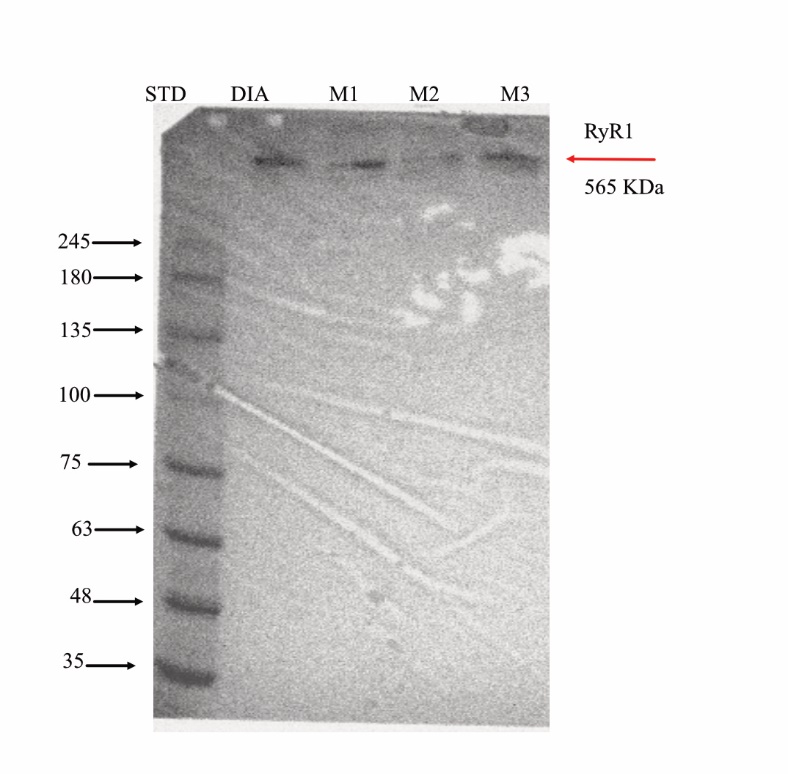


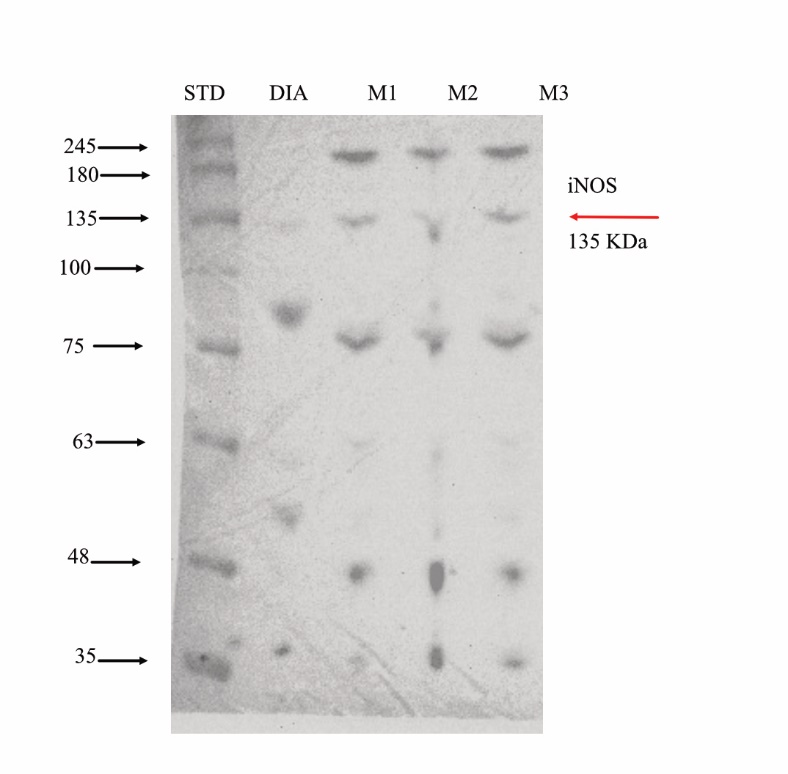

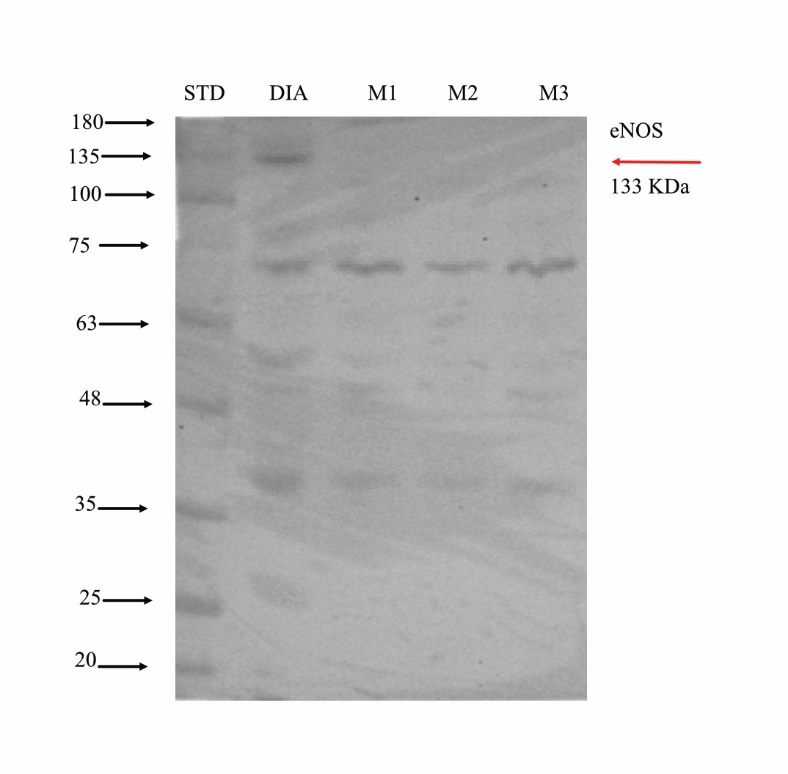

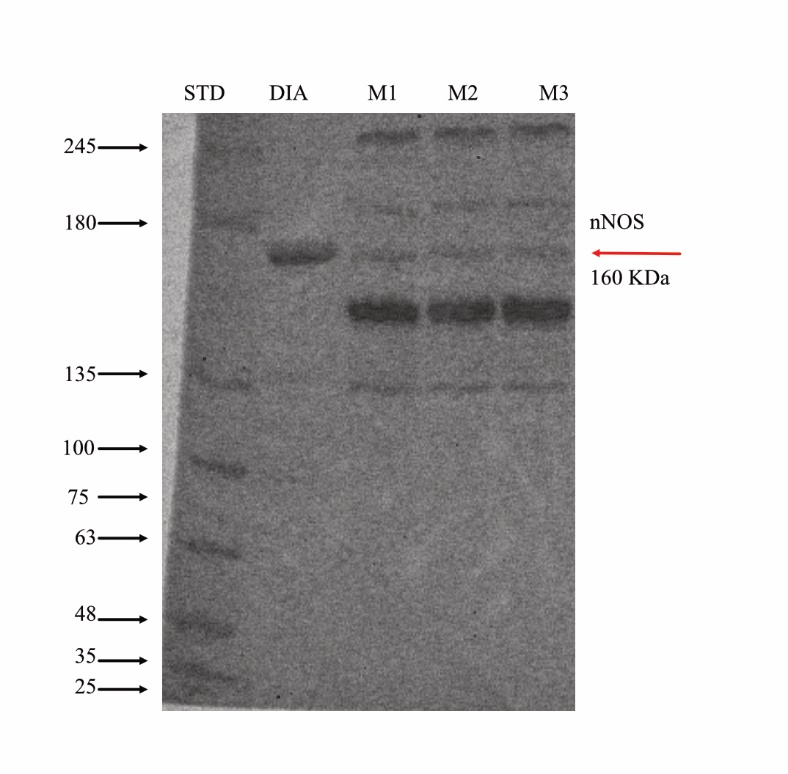

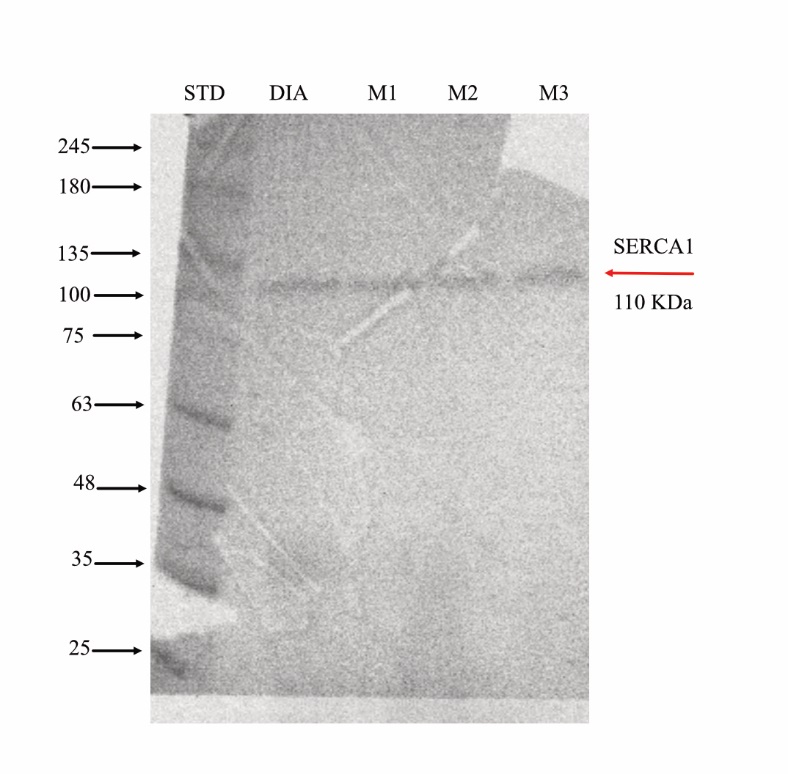


# Supplementary Tables

**Supplementary Table 1.** Force-Frequency data from diaphragm strip and myooid. Data normalized from 1 Hz response. The “n” corresponds to the number of mice or the number of myooids.

|  | Diaphragm | | | Myooid | | |
| --- | --- | --- | --- | --- | --- | --- |
| Frequency | Force | SD | n | Force | SD | n |
| 1 | 100.00 | 0.00 | 6 | 100.00 | 0.00 | 20 |
| 5 | 121.97 | 31.89 | 6 | 143.08 | 27.24 | 20 |
| 10 | 155.79 | 41.99 | 6 | 175.39 | 38.63 | 20 |
| 15 | 217.85 | 43.33 | 6 | 185.57 | 50.60 | 20 |
| 20 | 239.59 | 48.49 | 6 | 194.97 | 60.03 | 20 |
| 25 | 289.49 | 78.09 | 6 | 195.20 | 79.16 | 20 |
| 30 | 293.16 | 105.99 | 6 | 199.33 | 87.80 | 20 |
| 35 | 314.75 | 109.62 | 6 | 204.87 | 95.96 | 20 |
| 40 | 315.29 | 131.88 | 6 | 204.40 | 95.93 | 20 |
| 45 | 328.73 | 116.92 | 6 | 193.88 | 91.55 | 20 |
| 50 | 329.42 | 116.31 | 6 | 199.91 | 94.30 | 20 |
| 60 | 332.15 | 117.76 | 6 | 194.08 | 99.53 | 20 |

**Supplementary Table 2.** Effect of NO on the diaphragm’s response to subthreshold stimulation. Parameters analyzed were rheobase (V) and chronaxie (ms). The “n” corresponds to the number of mice.

| Rheobase | Control | SNAP | L-NAME |  | Chronaxie | Control | SNAP | L-NAME |
| --- | --- | --- | --- | --- | --- | --- | --- | --- |
| Mean | 15.08 | 11.00 | 18.54 |  | Mean | 1.67 | 0.68 | 1.58 |
| SD | 1.59 | 2.72 | 2.79 |  | SD | 0.41 | 0.49 | 0.49 |
| n | 6 | 6 | 6 |  | n | 6 | 6 | 6 |
| p-value |  | 0.0107 | 0.0262 |  | p-value |  | 0.0036 | ns |

**Supplementary Table 3.** Analysis of SNAP and L-NAME on the response of diaphragm to force-frequency protocol. Force data in KN/m^2^ normalized to the diaphragm’s CSA. The “n” corresponds to the number of mice.

|  | Control | | | SNAP | | | L-NAME | | |
| --- | --- | --- | --- | --- | --- | --- | --- | --- | --- |
| Frequency | Force | SD | n | Force | SD | n | Force | SD | n |
| 1 | 34.63 | 13.76 | 6 | 10.12 | 6.10 | 6 | 63.61 | 12.74 | 6 |
| 5 | 44.48 | 17.36 | 6 | 12.85 | 8.99 | 6 | 76.56 | 16.76 | 6 |
| 10 | 51.58 | 21.45 | 6 | 17.33 | 6.99 | 6 | 106.67 | 31.95 | 6 |
| 15 | 73.90 | 20.22 | 6 | 23.67 | 11.61 | 6 | 117.44 | 32.18 | 6 |
| 20 | 81.65 | 21.21 | 6 | 33.35 | 12.88 | 6 | 145.01 | 40.11 | 6 |
| 25 | 92.43 | 22.53 | 6 | 38.34 | 18.92 | 6 | 165.17 | 48.78 | 6 |
| 30 | 98.42 | 38.28 | 6 | 59.61 | 14.45 | 6 | 171.35 | 52.93 | 6 |
| 35 | 111.94 | 18.89 | 6 | 69.69 | 20.97 | 6 | 174.25 | 51.61 | 6 |
| 40 | 118.89 | 20.93 | 6 | 86.62 | 19.35 | 6 | 179.26 | 48.21 | 6 |
| 45 | 123.31 | 18.94 | 6 | 95.20 | 23.97 | 6 | 182.26 | 45.46 | 6 |
| 50 | 129.06 | 22.45 | 6 | 107.69 | 25.78 | 6 | 184.00 | 44.65 | 6 |
| 60 | 141.50 | 27.28 | 6 | 116.27 | 27.72 | 6 | 185.15 | 46.49 | 6 |
| Interaction (Frequency X Treatment) F(_22, 176_) = 2.652; p< 0.001 | | | | | | |  |  |  |

**Supplementary Table 4.** Analyses of SNAP and L-NAME on rheobase (V) and chronaxie (ms) of the myooid’s response to subthreshold stimulation. The “n” corresponds to the number of myooids.

| Rheobase | Control | SNAP | L-NAME |  | Chronaxie | Control | SNAP | L-NAME |
| --- | --- | --- | --- | --- | --- | --- | --- | --- |
| Mean | 9.15 | 6.15 | 12.65 |  | Mean | 1.48 | 1.47 | 1.75 |
| SD | 4.01 | 2.72 | 4.69 |  | SD | 1.08 | 0.59 | 1.39 |
| n | 20 | 20 | 20 |  | n | 20 | 20 | 20 |
| p-value |  | 0.0358 | 0.0123 |  | p-value |  | ns | ns |

**Supplementary Table 5.** Analysis of SNAP and L-NAME on the response of myooid to force-frequency protocol. Force data in mN/m^2^ normalized to the myooid’s CSA. The “n” corresponds to the number of myooids.

|  | Control | | | SNAP | | | L-NAME | | |
| --- | --- | --- | --- | --- | --- | --- | --- | --- | --- |
| Frequency | Force | SD | n | Force | SD | n | Force | SD | n |
| 1 | 4.50 | 0.86 | 20 | 2.64 | 0.50 | 20 | 8.26 | 2.19 | 20 |
| 5 | 6.11 | 1.11 | 20 | 3.80 | 0.75 | 20 | 12.23 | 3.73 | 20 |
| 10 | 7.65 | 1.38 | 20 | 4.37 | 0.88 | 20 | 15.34 | 5.02 | 20 |
| 15 | 8.33 | 1.58 | 20 | 4.79 | 0.95 | 20 | 16.11 | 5.47 | 20 |
| 20 | 8.83 | 1.77 | 20 | 5.02 | 0.97 | 20 | 16.62 | 5.70 | 20 |
| 25 | 9.00 | 1.89 | 20 | 5.33 | 1.04 | 20 | 16.38 | 5.85 | 20 |
| 30 | 9.29 | 2.00 | 20 | 5.46 | 1.06 | 20 | 16.65 | 5.87 | 20 |
| 35 | 9.64 | 2.14 | 20 | 5.57 | 1.09 | 20 | 16.66 | 5.85 | 20 |
| 40 | 9.73 | 2.18 | 20 | 5.56 | 1.05 | 20 | 16.01 | 5.78 | 20 |
| 45 | 9.43 | 2.12 | 20 | 5.56 | 1.08 | 20 | 15.36 | 5.14 | 20 |
| 50 | 9.17 | 1.87 | 20 | 5.31 | 1.00 | 20 | 16.35 | 6.53 | 20 |
| 60 | 8.95 | 1.87 | 20 | 5.15 | 0.93 | 20 | 14.19 | 4.79 | 20 |
| Interaction (Frequency X Treatment) F(_22,352_) = 3.378; p < 0.001 | | | | | |  |  |  |  |

**Supplementary Table 6.** Analyses of biophysical parameters response of diaphragm strips and myooids to 1Hz (single twitch) electrical stimulation.

|  | Diaphragm | | | Myooid | | |
| --- | --- | --- | --- | --- | --- | --- |
|  | Control | SNAP | L-NAME | Control | SNAP | L-NAME |
|  | Peak (kN/m^2^) | | | Peak (mN/m^2^) | | |
| Mean | 58.00 | 34.44 | 77.35 | 5.44 | 2.86 | 8.70 |
| SD | 18.73 | 8.23 | 6.36 | 1.52 | 1.20 | 2.81 |
| p-value |  | 0.0042 | 0.0168 |  | <0.001 | <0.001 |
|  |  |  |  |  |  |  |
|  | Time to Peak (ms) | | | Time to Peak (ms) | | |
| Mean | 43.29 | 35.68 | 30.82 | 102.50 | 75.00 | 101.20 |
| SD | 8.21 | 10.20 | 7.20 | 39.68 | 20.50 | 25.69 |
| p-value |  | ns | 0.0269 |  | 0.012 | ns |
|  |  |  |  |  |  |  |
|  | Duration (ms) | | | Duration (ms) | | |
| Mean | 289.30 | 228.50 | 173.80 | 722.10 | 876.60 | 772.40 |
| SD | 109.30 | 71.61 | 75.07 | 521.90 | 590.10 | 345.90 |
| p-value |  | ns | 0.042 |  | ns | ns |
|  |  |  |  |  |  |  |
|  | Area (mN*s) | | | Area (µN*s) | | |
| Mean | 7.45 | 2.86 | 5.24 | 1.40 | 1.11 | 2.69 |
| SD | 3.68 | 0.98 | 1.71 | 0.68 | 1.03 | 0.86 |
| p-value |  | 0.0042 | ns |  | ns | <0.001 |
|  |  |  |  |  |  |  |
|  | Duration_50_ (ms) | | | Duration_50_ (ms) | | |
| Mean | 99.86 | 74.25 | 64.36 | 168.20 | 204.10 | 200.40 |
| SD | 17.90 | 22.17 | 22.26 | 56.26 | 65.51 | 51.31 |
| p-value |  | ns | 0.0097 |  | ns | ns |
|  |  |  |  |  |  |  |
|  | Slope (N/s) | | | Slope (µN/s) | | |
| Mean | 1898.00 | 1206.00 | 2700.00 | 88.07 | 46.30 | 142.40 |
| SD | 609.70 | 357.50 | 449.40 | 21.41 | 22.18 | 61.14 |
| p-value |  | 0.0284 | 0.0114 |  | 0.0036 | <0.001 |

**Supplementary Table 7.** Analyses of Bay41-2272 and ODQ on rheobase (V) and chronaxie (ms) of the myooid’s response to subthreshold stimulation. The “n” corresponds to the number of myooids.

| Rheobase | Control | Bay41-2272 | ODQ |  | Chronaxie | Control | Bay41-2272 | ODQ |
| --- | --- | --- | --- | --- | --- | --- | --- | --- |
| Mean | 11.75 | 12.25 | 11.75 |  | Mean | 1.20 | 1.20 | 1.75 |
| SD | 2.90 | 1.71 | 2.00 |  | SD | 0.75 | 0.71 | 0.95 |
| n | 10 | 10 | 10 |  | n | 10 | 10 | 10 |
| p-value |  | ns | ns |  | p-value |  | ns | ns |

**Supplementary Table 8.** Analysis of Bay41-2272 and ODQ on the response of the myooids to force-frequency protocol. Force data in mN/m^2^ normalized to the myooid’s CSA. The “n” corresponds to the number of myooids.

|  | Control | | | Bay41-2272 | | | ODQ | | |
| --- | --- | --- | --- | --- | --- | --- | --- | --- | --- |
| Frequency | Force | SD | n | Force | SD | n | Force | SD | n |
| 1 | 5.78 | 1.01 | 10 | 3.46 | 1.30 | 10 | 9.59 | 1.74 | 10 |
| 5 | 8.49 | 2.92 | 10 | 4.46 | 1.79 | 10 | 13.23 | 3.24 | 10 |
| 10 | 8.61 | 2.43 | 10 | 4.41 | 2.12 | 10 | 14.24 | 4.40 | 10 |
| 15 | 8.80 | 2.71 | 10 | 5.01 | 1.98 | 10 | 14.01 | 4.92 | 10 |
| 20 | 9.07 | 2.90 | 10 | 5.27 | 2.44 | 10 | 15.45 | 5.68 | 10 |
| 25 | 8.52 | 2.02 | 10 | 4.92 | 2.16 | 10 | 15.05 | 5.95 | 10 |
| 30 | 8.48 | 2.09 | 10 | 5.48 | 2.61 | 10 | 16.07 | 5.47 | 10 |
| 35 | 9.22 | 2.71 | 10 | 5.29 | 2.56 | 10 | 15.61 | 5.24 | 10 |
| 40 | 8.74 | 2.70 | 10 | 5.51 | 1.93 | 10 | 15.07 | 5.68 | 10 |
| 45 | 8.43 | 2.91 | 10 | 5.45 | 2.09 | 10 | 15.79 | 6.51 | 10 |
| 50 | 8.56 | 2.36 | 10 | 5.33 | 2.00 | 10 | 14.68 | 5.11 | 10 |
| 60 | 8.64 | 2.23 | 10 | 4.74 | 2.03 | 10 | 14.75 | 5.28 | 10 |
| Interaction (Frequency X Treatment) F(_22, 297_) = 2.643; p < 0.001 | | | | | | |  |  |  |

**Supplementary Table 9.** Analyses of biophysical parameters response of Bay41-2272 and ODQ on myooids response to 1Hz (single twitch) electrical stimulation

|  | Myooid | | |
| --- | --- | --- | --- |
|  | Control | Bay41-2272 | ODQ |
|  | Peak (mN/m^2^) | | |
| Mean | 5.29 | 4.14 | 8.13 |
| SD | 1.04 | 1.18 | 1.42 |
| p-value |  | ns | <0.001 |
|  | | | |
|  | Time to Peak (ms) | | |
| Mean | 81.20 | 70.50 | 97.10 |
| SD | 19.15 | 34.67 | 42.35 |
| p-value |  | ns | ns |
|  | | | |
|  | Duration (ms) | | |
| Mean | 526.70 | 446.80 | 685.80 |
| SD | 260.20 | 250.30 | 435.80 |
| p-value |  | ns | ns |
|  | | | |
|  | Area (µN*s) | | |
| Mean | 1.45 | 0.69 | 2.41 |
| SD | 0.85 | 0.46 | 1.15 |
| p-value |  | ns | 0.0361 |
|  | | | |
|  | Duration_50_ (ms) | | |
| Mean | 162.50 | 175.60 | 202.40 |
| SD | 42.86 | 93.81 | 63.28 |
| p-value |  | ns | ns |
|  | | | |
|  | Slope (µN/s) | | |
| Mean | 96.69 | 68.38 | 140.00 |
| SD | 21.61 | 23.25 | 48.04 |
| p-value |  | ns | 0.0142 |

**Supplementary Table 10.** Analyses of 8pCPT and DT-3 on rheobase (V) and chronaxie (ms) of the myooid’s response to subthreshold stimulation. The “n” corresponds to the number of myooids.

| Rheobase | Control | 8pCPT | DT-3 |  | Chronaxie | Control | 8pCPT | DT-3 |
| --- | --- | --- | --- | --- | --- | --- | --- | --- |
| Mean | 15.35 | 17.75 | 18.50 |  | Mean | 1.15 | 0.70 | 1.30 |
| SD | 3.67 | 5.83 | 7.47 |  | SD | 0.71 | 0.35 | 0.95 |
| n | 10 | 10 | 10 |  | n | 10 | 10 | 10 |
| p-value |  | ns | ns |  | p-value |  | ns | ns |

**Supplementary Table 11.** Analysis of 8pCPT and DT-3 on the response of the myooids to force-frequency protocol. Force data in mN/m^2^ normalized to the myooid’s CSA. The “n” corresponds to the number of myooids.

|  | Control | | | 8pCPT | | | DT-3 | | |
| --- | --- | --- | --- | --- | --- | --- | --- | --- | --- |
| Frequency | Force | SD | n | Force | SD | n | Force | SD | n |
| 1 | 6.56 | 0.75 | 10 | 4.14 | 0.94 | 10 | 10.84 | 2.00 | 10 |
| 5 | 8.29 | 1.25 | 10 | 4.80 | 1.40 | 10 | 12.88 | 2.50 | 10 |
| 10 | 8.49 | 1.38 | 10 | 5.01 | 1.34 | 10 | 14.35 | 3.33 | 10 |
| 15 | 8.52 | 1.35 | 10 | 5.19 | 1.45 | 10 | 14.08 | 2.91 | 10 |
| 20 | 8.77 | 1.02 | 10 | 5.17 | 1.49 | 10 | 14.40 | 2.02 | 10 |
| 25 | 8.65 | 1.10 | 10 | 5.32 | 1.36 | 10 | 13.98 | 2.57 | 10 |
| 30 | 8.83 | 1.45 | 10 | 5.26 | 1.42 | 10 | 14.02 | 3.33 | 10 |
| 35 | 9.12 | 1.30 | 10 | 5.30 | 1.53 | 10 | 14.26 | 2.60 | 10 |
| 40 | 8.83 | 1.58 | 10 | 5.29 | 1.51 | 10 | 13.79 | 2.68 | 10 |
| 45 | 9.09 | 1.72 | 10 | 5.32 | 1.48 | 10 | 13.64 | 2.78 | 10 |
| 50 | 8.83 | 1.61 | 10 | 5.25 | 1.29 | 10 | 13.75 | 2.38 | 10 |
| 60 | 9.22 | 1.53 | 10 | 5.19 | 1.19 | 10 | 14.07 | 2.55 | 10 |
| Interaction (Frequency X Treatment) F(_22,297_) = 2.639, p< 0.001 | | | | | |  |  |  |  |

**Supplementary Table 12.** Analyses of biophysical parameters response of 8pCPT and DT-3on myooids response to 1Hz (single twitch) electrical stimulation

|  | Myooid | | |
| --- | --- | --- | --- |
|  | Control | 8pCPT | DT-3 |
|  | Peak (mN/m2) | | |
| Mean | 5.76 | 3.55 | 9.33 |
| SD | 0.78 | 0.85 | 1.94 |
| p-value |  | 0.0015 | <0.001 |
|  | | | |
|  | Time to Peak (ms) | | |
| Mean | 91.20 | 85.40 | 68.00 |
| SD | 16.44 | 21.94 | 15.43 |
| p-value |  | ns | 0.0153 |
|  | | | |
|  | Duration (ms) | | |
| Mean | 517.80 | 528.50 | 426.90 |
| SD | 174.70 | 158.90 | 216.50 |
| p-value |  | ns | ns |
|  | | | |
|  | Area (µN*s) | | |
| Mean | 1.09 | 0.72 | 1.57 |
| SD | 0.23 | 0.16 | 0.53 |
| p-value |  | 0.0417 | 0.0083 |
|  | | | |
|  | Duration50 (ms) | | |
| Mean | 166.10 | 170.60 | 141.40 |
| SD | 42.21 | 46.34 | 37.37 |
| p-value |  | ns | ns |
|  | | | |
|  | Slope (µN/s) | | |
| Mean | 105.90 | 64.37 | 236.60 |
| SD | 21.31 | 21.58 | 48.30 |
| p-value |  | 0.0167 | <0.001 |

**Supplementary Table 13.** Analyses of NEM and AA on rheobase (V) and chronaxie (ms) of the myooid’s response to subthreshold stimulation. The “n” corresponds to the number of myooids.

| Rheobase | Control | NEM | AA |  | Chronaxie | Control | NEM | AA |
| --- | --- | --- | --- | --- | --- | --- | --- | --- |
| Mean | 14.05 | 21.00 | 24.00 |  | Mean | 1.05 | 1.80 | 1.85 |
| SD | 2.83 | 6.15 | 4.60 |  | SD | 0.55 | 0.59 | 0.71 |
| n | 10 | 10 | 10 |  | n | 10 | 10 | 10 |
| p-value |  | 0.0028 | 0.001 |  | p-value |  | 0.0216 | 0.0142 |

**Supplementary Table 14.** Analysis of NEM and AA on the force-frequency protocol of the myooid. Force data in mN/m^2^ normalized to the myooid’s CSA. The “n” corresponds to the number of myooids.

|  | Control | | | 8pCPT | | | DT-3 | | |
| --- | --- | --- | --- | --- | --- | --- | --- | --- | --- |
| Frequency | Force | SD | n | Force | SD | n | Force | SD | n |
| 1 | 5.36 | 0.94 | 10 | 5.85 | 1.35 | 10 | 5.80 | 1.62 | 10 |
| 5 | 6.47 | 1.51 | 10 | 6.83 | 1.93 | 10 | 8.72 | 2.58 | 10 |
| 10 | 7.35 | 3.33 | 10 | 7.59 | 3.80 | 10 | 9.23 | 3.54 | 10 |
| 15 | 7.36 | 2.53 | 10 | 7.42 | 2.63 | 10 | 9.11 | 3.48 | 10 |
| 20 | 7.51 | 2.88 | 10 | 7.65 | 2.89 | 10 | 8.92 | 3.65 | 10 |
| 25 | 7.65 | 2.78 | 10 | 7.72 | 2.25 | 10 | 8.40 | 3.38 | 10 |
| 30 | 7.52 | 2.57 | 10 | 7.58 | 1.66 | 10 | 9.00 | 3.76 | 10 |
| 35 | 8.04 | 2.93 | 10 | 8.05 | 2.00 | 10 | 7.97 | 3.42 | 10 |
| 40 | 8.28 | 3.42 | 10 | 7.59 | 1.83 | 10 | 7.87 | 3.82 | 10 |
| 45 | 8.15 | 3.97 | 10 | 7.68 | 2.24 | 10 | 8.12 | 4.15 | 10 |
| 50 | 8.15 | 4.15 | 10 | 7.67 | 2.10 | 10 | 8.01 | 3.66 | 10 |
| 60 | 8.20 | 4.40 | 10 | 7.15 | 2.21 | 10 | 7.84 | 3.14 | 10 |
| Treatment F(_2,27_) = 0.3298, p= 0.7219 | | | | |  |  |  |  |  |

**Supplementary Table 15.** Analyses of biophysical parameters response of NEM and AA on myooids response to 1Hz (single twitch) electrical stimulation.

|  | Myooid | | |
| --- | --- | --- | --- |
|  | Control | NEM | AA |
|  | Peak (mN/m2) | | |
| Mean | 5.06 | 5.72 | 5.09 |
| SD | 1.16 | 1.76 | 1.58 |
| p-value |  | ns | ns |
|  | | | |
|  | Time to Peak (ms) | | |
| Mean | 100.50 | 95.10 | 119.80 |
| SD | 11.18 | 9.45 | 30.29 |
| p-value |  | ns | ns |
|  | | | |
|  | Duration (ms) | | |
| Mean | 729.60 | 784.10 | 870.20 |
| SD | 393.40 | 384.60 | 285.40 |
| p-value |  | ns | ns |
|  | | | |
|  | Area (µN*s) | | |
| Mean | 1.37 | 1.67 | 2.16 |
| SD | 0.42 | 0.71 | 1.14 |
| p-value |  | ns | ns |
|  | | | |
|  | Duration50 (ms) | | |
| Mean | 221.40 | 222.60 | 302.00 |
| SD | 105.00 | 104.30 | 173.10 |
| p-value |  | ns | ns |
|  | | | |
|  | Slope (µN/s) | | |
| Mean | 80.24 | 93.01 | 77.60 |
| SD | 30.53 | 34.21 | 29.13 |
| p-value |  | ns | ns |

**Supplementary Table 16.** Analyses of SMTC and 1400W on rheobase (V) and chronaxie (ms) of the myooid’s response to subthreshold stimulation. The “n” corresponds to the number of myooids.

| Rheobase | Control | SMTC | 1400W |  | Chronaxie | Control | SMTC | 1400W |
| --- | --- | --- | --- | --- | --- | --- | --- | --- |
| Mean | 14.90 | 14.10 | 23.20 |  | Mean | 1.25 | 1.70 | 1.70 |
| SD | 3.34 | 3.64 | 5.88 |  | SD | 0.54 | 0.35 | 0.54 |
| n | 10 | 10 | 10 |  | n | 10 | 10 | 10 |
| p-value |  | ns | 0.001 |  | p-value |  | ns | ns |

**Supplementary Table 17.** Analysis of SMTC and 1400W on the force-frequency protocol of the myooid. Force data in mN/m^2^ normalized to myooid’s CSA. The “n” corresponds to the number of myooids.

|  | Control | | | SMTC | | | 1400W | | |
| --- | --- | --- | --- | --- | --- | --- | --- | --- | --- |
| Frequency | Force | SD | n | Force | SD | n | Force | SD | n |
| 1 | 5.75 | 1.04 | 10 | 10.83 | 2.98 | 10 | 6.33 | 2.13 | 10 |
| 5 | 8.14 | 3.14 | 10 | 13.26 | 3.50 | 10 | 7.70 | 2.38 | 10 |
| 10 | 9.67 | 3.28 | 10 | 14.57 | 4.78 | 10 | 8.09 | 3.81 | 10 |
| 15 | 9.59 | 2.66 | 10 | 14.46 | 5.28 | 10 | 7.95 | 2.54 | 10 |
| 20 | 9.12 | 3.83 | 10 | 14.73 | 6.10 | 10 | 9.14 | 2.45 | 10 |
| 25 | 8.90 | 2.92 | 10 | 14.81 | 6.44 | 10 | 9.59 | 2.99 | 10 |
| 30 | 8.67 | 2.91 | 10 | 15.39 | 6.58 | 10 | 10.28 | 3.81 | 10 |
| 35 | 9.38 | 3.68 | 10 | 14.70 | 6.44 | 10 | 10.63 | 3.83 | 10 |
| 40 | 9.61 | 3.96 | 10 | 14.33 | 6.64 | 10 | 10.02 | 3.41 | 10 |
| 45 | 9.50 | 4.28 | 10 | 14.20 | 6.61 | 10 | 10.41 | 3.55 | 10 |
| 50 | 9.60 | 4.16 | 10 | 14.15 | 5.70 | 10 | 10.16 | 3.51 | 10 |
| 60 | 9.60 | 4.36 | 10 | 14.41 | 6.16 | 10 | 10.17 | 3.21 | 10 |
| Treatment F(_2,27_) = 5.925; p< 0.0074 | | | | |  |  |  |  |  |

**Supplementary Table 18.** Analyses of biophysical parameters response of SMTC and 1400W on myooids response to 1Hz (single twitch) electrical stimulation.

|  | Myooid | | |
| --- | --- | --- | --- |
|  | Control | SMTC | 1400W |
|  | Peak (mN/m2) | | |
| Mean | 5.37 | 10.14 | 4.79 |
| SD | 1.17 | 1.32 | 1.52 |
| p-value |  | <0.001 | ns |
|  | | | |
|  | Time to Peak (ms) | | |
| Mean | 113.00 | 84.80 | 111.00 |
| SD | 30.94 | 19.43 | 24.33 |
| p-value |  | 0.0357 | ns |
|  | | | |
|  | Duration (ms) | | |
| Mean | 662.50 | 800.60 | 1047.00 |
| SD | 343.70 | 426.70 | 478.20 |
| p-value |  | ns | ns |
|  | | | |
|  | Area (µN*s) | | |
| Mean | 1.44 | 2.44 | 1.34 |
| SD | 0.38 | 0.71 | 0.35 |
| p-value |  | <0.001 | ns |
|  | | | |
|  | Duration50 (ms) | | |
| Mean | 177.50 | 182.90 | 192.10 |
| SD | 47.70 | 37.26 | 40.45 |
| p-value |  | ns | ns |
|  | | | |
|  | Slope (µN/s) | | |
| Mean | 100.50 | 154.40 | 87.91 |
| SD | 18.71 | 24.38 | 32.41 |
| p-value |  | <0.001 | ns |
|  |  |  |  |
|  |  |  |  |
